# Neurofilament light interaction with GluN1 modulates neurotransmission and schizophrenia-associated behaviors

**DOI:** 10.1038/s41398-018-0194-7

**Published:** 2018-08-24

**Authors:** Aidong Yuan, Veeranna, Henry Sershen, Balapal S. Basavarajappa, John F. Smiley, Audrey Hashim, Cynthia Bleiwas, Martin Berg, David N. Guifoyle, Shivakumar Subbanna, Sandipkumar Darji, Asok Kumar, Mala V. Rao, Donald A. Wilson, Jean-Pierre Julien, Daniel C. Javitt, Ralph A. Nixon

**Affiliations:** 1 Center for Dementia Research, Nathan Kline Institute, Orangeburg, NY 10962 USA; 2 Departments of Psychiatry, New York University School of Medicine, New York, NY 10016 USA; 3 Neurochemistry Division, Nathan Kline Institute, Orangeburg, NY 10962 USA; 4 Analytical Psychopharmacology Division, Nathan Kline Institute, Orangeburg, NY 10962 USA; 5 Department of Psychiatry, College of Physicians & Surgeons, Columbia University, New York, NY 10032 USA; 6 New York State Psychiatric Institute, New York, NY 10032 USA; 7 Center for Biomedical Imaging and Neuromodulation, Nathan Kline Institute, Orangeburg, NY 10962 USA; 8 Emotional Brain Institute, Nathan Kline Institute, Orangeburg, NY 10962 USA; 9 Child and Adolescent Psychiatry, New York University School of Medicine, New York, NY 10016 USA; 10 Neuroscience Institute, New York University School of Medicine, New York, NY 10016 USA; 11 Centre de Recherche du Centre Hospitalier de l’Université Laval, Département d’anatomie et physiologie de l’Université Laval, 2795 boul. Laurier, Québec, G1V 4G2 Canada; 12 Schizophrenia Research, Nathan Kline Institute, Orangeburg, NY 10962 USA; 13 Department of Cell Biology, New York University School of Medicine, New York, NY 10016 USA

## Abstract

Neurofilament (NFL) proteins have recently been found to play unique roles in synapses. NFL is known to interact with the GluN1 subunit of *N*-methyl-d-aspartic acid (NMDAR) and be reduced in schizophrenia though functional consequences are unknown. Here we investigated whether the interaction of NFL with GluN1 modulates synaptic transmission and schizophrenia-associated behaviors. The interaction of NFL with GluN1 was assessed by means of molecular, pharmacological, electrophysiological, magnetic resonance spectroscopy (MRS), and schizophrenia-associated behavior analyses. NFL deficits cause an NMDAR hypofunction phenotype including abnormal hippocampal function, as seen in schizophrenia. NFL−/− deletion in mice reduces dendritic spines and GluN1 protein levels, elevates ubiquitin-dependent turnover of GluN1 and hippocampal glutamate measured by MRS, and depresses hippocampal long-term potentiation. NMDAR-related behaviors are also impaired, including pup retrieval, spatial and social memory, prepulse inhibition, night-time activity, and response to NMDAR antagonist, whereas motor deficits are minimal. Importantly, partially lowering NFL in NFL+/− mice to levels seen regionally in schizophrenia, induced similar but milder NMDAR-related synaptic and behavioral deficits. Our findings support an emerging view that central nervous system neurofilament subunits including NFL in the present report, serve distinctive, critical roles in synapses relevant to neuropsychiatric diseases.

## Introduction

Neurofilaments (NF), unlike intermediate filaments of non-neuronal cells, are composed of four distinct subunits under complex regulation by phosphorylation^1^. The purpose of this added complexity of neuronal intermediate filaments has, until recently, been a puzzle. As linear heteropolymers, neurofilaments support the radial expansion of large myelinated axons. Because rare mutations of NFL (neurofilament light subunit, *NEFL*) cause a subtype of the peripheral axonopathy, Charcot-Marie-Tooth disease^2–4^, attention has previously focused mainly on roles of NF proteins in maintaining the elaborate axonal NF lattice of large caliber peripheral axons. In the central nervous system (CNS), however, NFs play a minor role in axon caliber expansion^5,6^ and recent evidence has shown that NF proteins form distinct protofilamentous assemblies within synaptic spines where the individual NF subunits differentially interact with specific neurotransmitter receptors and modulate their activity^7^.

Dendritic spines are believed to be an anatomical substrate for memory storage and synaptic transmission^8,9^ and a key feature of these spines is the postsynaptic density (PSD), which is critical for receptor stability and activity. The PSD is anchored and its functions are regulated by an actin-based cytoskeletal scaffold^10^ composed of proteins that interact directly with receptors, such as *N*-methyl-d-aspartic acid (NMDAR)^11^. Notably, mutations or deletions of many of the genes encoding proteins comprising the synaptic scaffold are believed to cause neuropsychiatric disorders, including schizophrenia, autism, and mental retardation (Supplementary Table S1). NF proteins have rarely been considered in relation to the synaptic scaffold, however, the evidence for a significant synaptic location of NF proteins and a wealth of recent proteomic data led us to propose that NFP is a core synaptic scaffold component^6,7^ and that NFL, which is known to interact with the GluN1 subunit of NMDAR^12^, may be essential for the proper functioning of this receptor. Supporting this possibility, an NFL interactome constructed from published data (Supplementary Figure S1) reveals direct interactions of NFL with many of the known postsynaptic scaffold proteins. Notably, a high proportion of these proteins are known to be altered in schizophrenia and other neuropsychiatric diseases (Supplementary Table S1).

The NMDAR, composed of four subunits including the essential GluN1 subunit^13,14^, is located on the postsynaptic membrane where it contributes to the expression of long-term potentiation (LTP) and synaptic plasticity thought to underlie learning and memory^15^. NMDAR hypofunction is believed to be involved in the pathophysiology of schizophrenia and genetic, biochemical, and pharmacological evidence documents reduced expression of particular subunits of the receptor, including GluN1, in the brains of individuals with schizophrenia^16–21^. Mice expressing reduced levels of the GluN1 subunit have been proposed as an animal model of schizophrenia^22^. NMDAR is also a major component of the PSD complex and its GluN1 subunit interacts with NFL in vitro and in non-neuronal cells transfected with NFL protein^12^. Moreover, like the GluN1 deficit in schizophrenia, substantially reduced levels of NFL are a remarkably consistent finding in affected regions of schizophrenic brain (Supplementary Table S2) although the functional significance of these deficits is unclear. Also, although we previously showed that the NFL subunit is an integral component of synapses, its functions at this site have not been previously addressed.

In the present report, we establish novel synaptic roles for the NFL subunit in maintaining spine morphology, stabilizing GluN1 levels, and modulating NMDAR function and related behavior. NFL gene deletion in mice lowered numbers and lengths of dendritic spines, depressed hippocampal long-term potentiation induction, and selectively depressed NMDAR–GluN1 protein levels and NMDAR-related behaviors while adaptively raising hippocampal glutamate levels. Importantly, we observed a similar range of NMDAR-related synaptic and behavioral deficits, albeit milder than in NFL-null mice, in NFL+/− mice, in which brain NFL levels were lowered 40–50%, a reduction within a range of NFL deficits seen in brain regions implicated in schizophrenia^23^ (Supplementary Table S2). Our findings reinforce an emerging concept that CNS NFL serve distinctive roles in synaptic transmission and behaviors related to the modulation of specific neurotransmitter receptors and we identify specific roles of the NFL subunit in NMDAR function.

## Materials and methods

### Generation of mutant animals, drugs, and antibodies

Please see Supplementary Information.

### Analytical methods

Our published methods were used for all the procedures. Please see Supplementary Information.

## Results

### NFL is essential for the maintenance of dendritic spine structure and function

**Fig. 1:**
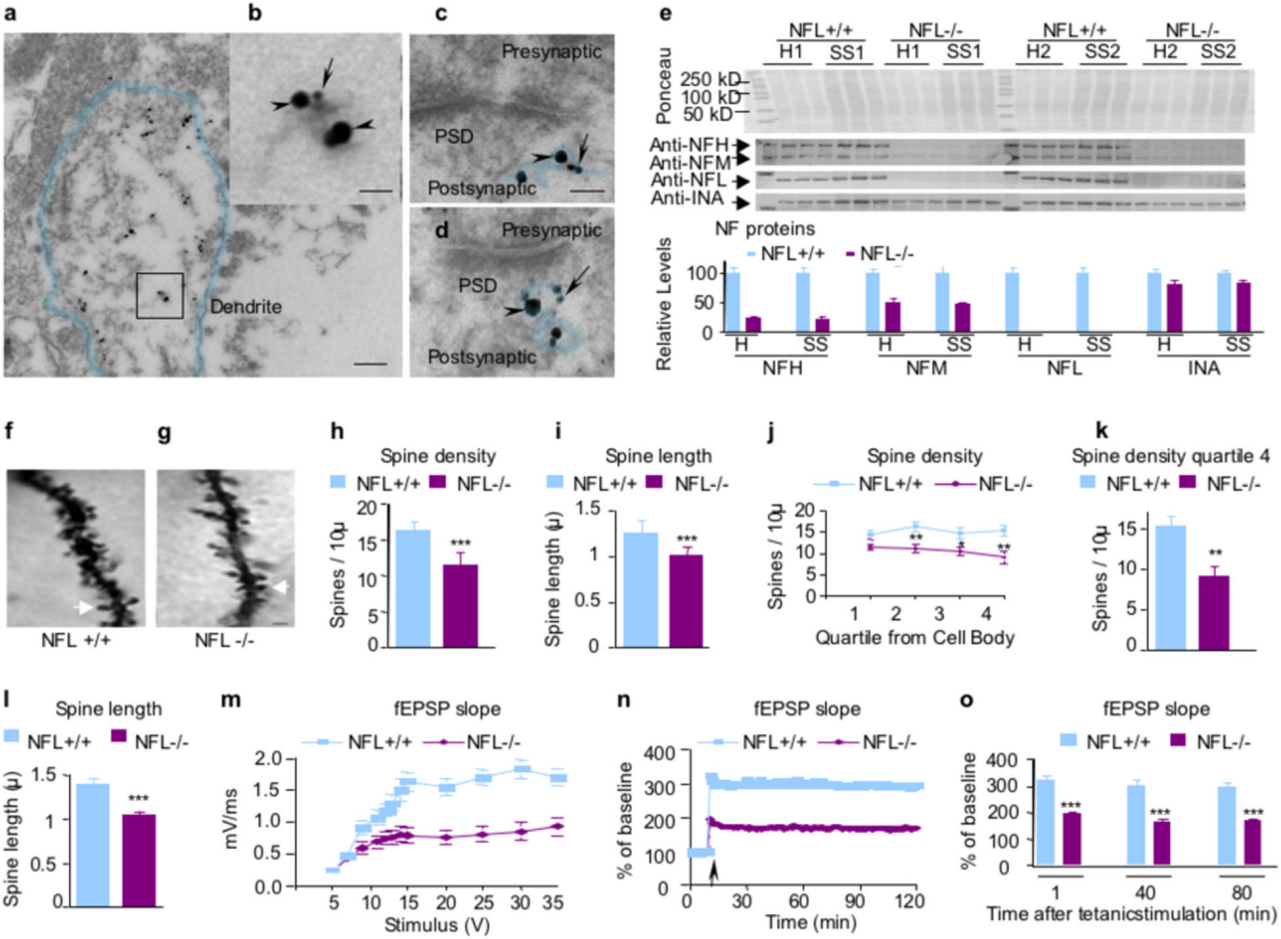
Reduced dendritic spine length and density and impaired CA1 LTP in the hippocampus of NFL−/− mice. Ultrastructural colocalization of NFL (arrowheads) and NFH (arrows) on the same NF within dendrites **a**, **b** and postsynaptic terminals **c**, **d** by immunogold electron microscopy. Dendrite boundaries and postsynaptic neurofilaments are outlined to clearly indicate the morphology. PSD: postsynaptic density. Scale bars, 150 nm in **a**; 30 nm in **b**; 40 nm in **c**, and **d**. Protein extracts prepared from total hippocampus homogenate and hippocampal synaptosomal fractions were separated on an 8.5% SDS-polyacrylamide gel and transferred to nitrocellulose membrane. Membranes were blotted with different antibodies. NFL were undetectable in NFL−/− mice **e**. NFH and alpha-internexin in NFL−/− mice were reduced to 22 and 82% of wild-type level, respectively, in both total homogenate and synaptosomes. NFM was reduced to 50% in total homogenate and 47% of wild-type level in synaptosomes, respectively. Data are expressed as Mean ± SEM, *n* = 6. H: homogenate; SS: synaptosomal fractions. Spine density was compared between NFL+/+ and NFL−/−. Spine density is expressed as the number of spines per 10 microns of dendrite. **f**, **g** Spine density on CA1 pyramidal neurons was reduced on dendrites in NFL−/− (*p* < 0.0001) **h**. Spine length is expressed as microns per spine. Spine length on CA1 pyramidal neurons was also reduced on dendrites in NFL−/− (*p* < 0.001) **i**. Data are expressed as mean ± SEM, *n* = 8. Arrows point to a representative spine in each genotype. Scale bar: 1 µm. Reduction of dendrite length (Supplementary Table S3), dendritic spine density **j**, **k** and spine length **l** in dentate gyrus granule cells of NFL−/− mice. **m** A summary graph showing the field I/O relationship for NFL+/+ (green) and NFL−/− (red) mice. **n** A time course of the average of the fEPSP slopes from slices obtained from NFL+/+ and NFL−/− mice. The fEPSP slopes were normalized to the average value during the 10 min prior to stimulation in each experiment. An arrow shows the time of tetanic stimulation (four pulses at 100 Hz with bursts repeated at 5 Hz, and each tetanus including three 10-burst trains separated by 15 s). **o** A combined plot of the averages of fEPSP slopes at several time points. Each point is the mean ± SEM (*n* = 7 mice per group, 14–20 slices per group). Two-way ANOVA with Bonferroni’s post hoc test; *p* < 0.001. *: *p* < 0.05; **: *p* < 0.01; ***: *p* < 0.001

Immuno-electron microscopy (EM) studies confirmed ultrastructural colocalization of NFL and NFH on the same filament within dendrites (Fig. 1a, b) and postsynaptic terminals (Fig. 1c, d). Densitometric quantification of immunostained proteins revealed that levels of NFL proteins in hippocampal homogenates and synaptosomes from NFL−/− mice were undetectable, as expected (Fig. 1e). Levels of NFH were reduced to 22% of normal wild-type levels. By contrast, synaptosomes in these mice retained ~ 50% of normal levels of NFM and 80% of normal INA, reflecting their known close physical and functional partnership^24^. Subunit stoichiometry in NFL−/− hippocampus is comparable to previous analyses of optic axons^25^. An earlier study reporting NFM and NFH in NFL−/− mice to be only 5% of WT levels^26^ can be explained by the fact that only phosphorylation-dependent isoforms were used in this study, whereas we used phosphorylation-independent anti-NFM and anti-NFH antibodies, which detect the larger total populations of each subunit.

We next used a rapid Golgi impregnation method to examine the effects of NFL deletion on the dendritic spines of hippocampal CA1 neurons (Fig. 1f, g). Spine density on dendritic arbors of CA1 neurons were significantly reduced in NFL−/− mice compared with NFL+/+ mice (*p* < 0.0001, *n* = 8) (Fig. 1h). Spine length was also significantly decreased (*p* < 0.001) (Fig. 1i). Similar changes in spine density and spine length were seen in dentate gyrus granule cells (Fig. 1j−l). In addition, path lengths of dendrites were significantly reduced in dentate gyrus granule cells of NFL−/− mice (Supplementary Table S3). NFL deletion had a greater effect on spines (lowered 41% in spine density and 24% in spine length) (Fig. 1k, l) than on dendrites (lowered 13% in average path) (Supplementary Table S3), further suggesting the important structural role of NF proteins specifically within synaptic spines. Given the changes in spine morphology, we investigated whether or not NFL−/− mice have LTP deficits. Hippocampal basal synaptic neurotransmission and LTP in the Schaffer collateral pathway of hippocampal slices were found significantly impaired in NFL−/− as compared with NFL+/+ mice (*p* < 0.001, *n* = 7) (Fig. 1m–o).

### NFL is essential for maintenance of NMDAR stability and activity at synapses

**Fig. 2:**
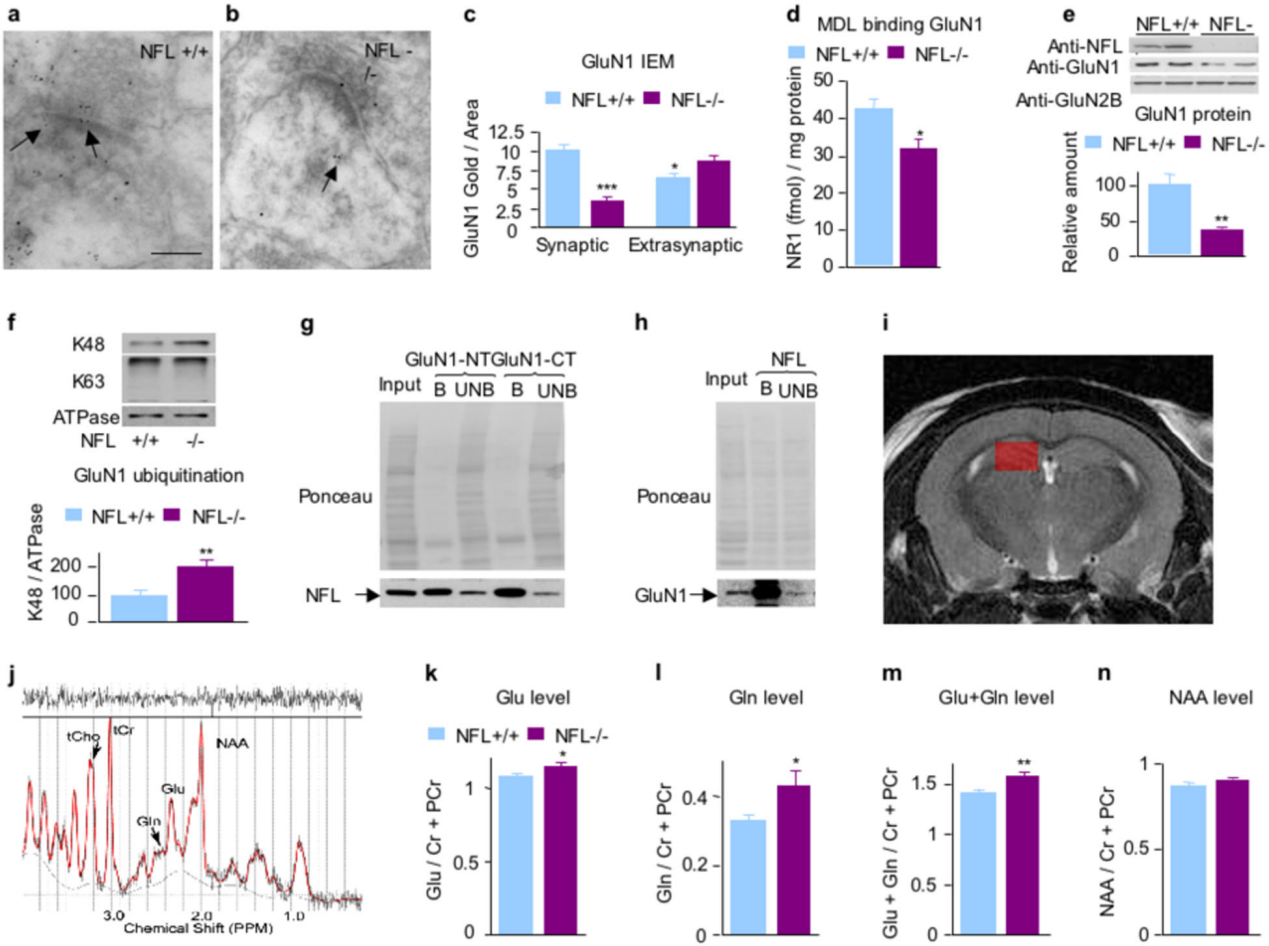
Reduced GluN1 expression and increased glutamate and glutamine levels in hippocampal ^1^H MRS metabolite measurements of NFL−/− mice as compared with NFL+/+ controls. In the absence of NFL, the level of NMDAR–GluN1 subunit in hippocampus was significantly decreased by immuno-EM with anti-GluN1 monoclonal antibody **a**–**c**. Scale bar in **a**, **b**: 150 nm. *: *p* < 0.05; ***: *p* < 0.001. Reduced level of GluN1 was confirmed by decreased binding of GluN1 subunit of NMDAR ligand [^3^H]MDL105,519 in the hippocampus of NFL−/− mice. *P* < 0.05, *n* = 8 **d**. Western blot with anti-GluN1 antibody also confirmed a reduced level of synaptosomal GluN1 from hippocampi, whereas the level of NR2b subunit was not significantly affected **e**. **f** Increased K48-linkage specific ubiquitin signals in GluN1-rich postsynaptic membrane fractions isolated from hippocampal synaptosomes of NFL−/− mice. GluN1-rich postsynaptic membrane fractions prepared from hippocampal synaptosomes were separated on an 8.5% SDS-polyacrylamide gel and transferred to nitrocellulose membrane. Membranes were blotted with K48-linkage-specific ubiquitin antibody and ATPase antibody. Densitometric quantification of immunostained proteins showed that K48-linkage-specific ubiquitin signals normalized to ATPase are significantly increased in the absence of NFL (*p* < 0.01, *n* = 8–12), whereas K63-linkage-specific ubiquitin signals was not significantly affected. **g**, **h** Co-immunoprecipitation of NFL with GluN1 from hippocampal synaptosomal preparations. GluN1-NT, GluN1 N-terminus antibody; GluN1-CT, GluN1 C-terminus antibody; B, bound; UB, unbound. *: *p* < 0.05; **: *p* < 0.01. **i** The VOI size was 5 µl (1 × 2 × 2.5 mm^3^) within hippocampus overlaid in red on the coronal anatomical scan. **j** Representative LCModel output spectrum from the hippocampus of a single NFL−/− mouse showing major labeled metabolites (*N*-Acetyl aspartate (NAA), glutamate (Glu), glutamine (Gln), total creatine (tCr), and total choline (tCho)). The black line represents the raw data and the red line represents the fit. The plot at the top shows the difference between plots. A good fit should look like random noise with oscillations about zero. **k** ^1^H MRS glutamate concentration in NFL−/− versus NFL+/+ mice in the hippocampus showed a significant difference. **l**, **m** ^1^H MRS glutamine and glutamate plus glutamine concentrations in NFL−/− versus NFL+/+ mice in the hippocampus also showed significant differences. **n** ^1^H MRS NAA concentration in NFL−/− versus NFL+/+ mice in the hippocampus did not show a significant difference. All data are presented as mean ± SEM (*n* = 16). *: *p* < 0.05; **: *p* < 0.01. Both female and male NFL−/− mice showed similar direction of change in glutamate and glutamine levels as compared with NFL+/+ mice (Supplementary Figure S3) and no significant differences of glutamate levels were observed between gender in NFL+/+ and NFL−/− mice (Supplementary Figure S4)

Immuno-EM studies with anti-GluN1 antibody revealed reduced GluN1 immunoreactivity in hippocampal synapses (Fig. 2a–c). [^3^H]MDL105,519 ligand binding to GluN1 was also significantly reduced in the hippocampal homogenates of NFL−/− as compared with NFL+/+ mice (Fig. 2d). Western blot studies showed that GluN1 subunit levels in hippocampal synaptosomes from NFL−/− mice were also substantially reduced (lowered 65%, *p* < 0.001) (Fig. 2e). Similar results were also observed in triple knockout mice lacking alpha-internexin, NFH, and NFL mice (Supplementary Figure S2). Densitometric quantification of immunostained proteins revealed that proteasome-degradation-related K48-linkage specific ubiquitin signals normalized to ATPase signal, a marker of plasma membrane^27^, are significantly increased in the absence of NFL (*p* < 0.05, mean ± SEM, *n* = 8–12) (Fig. 2f), whereas non-proteasome-degradation-related k63-linkage specific ubiquitin signals were not significantly altered^28^. In synaptosomal fractions, we also showed co-immunoprecipitation of GluN1 with NFL (Fig. 2g) and NFL with GluN1 (Fig. 2h), further substantiating in vivo interaction between GluN1 and NFL.

### Increased glutamate in the hippocampus of NFL−/− measured by ^1^H MRS

To determine whether lack of NFL affects glutamate level in hippocampus, we used in vivo magnetic resonance spectroscopy to measure regional content of relevant amino acids and metabolites in brain. The levels of Glu (*p* < 0.05, *n* = 16) and Gln (*p* < 0.01) concentrations were significantly increased in the hippocampal region of NFL−/− as compared with NFL mice (Fig. 2i–m). Hippocampal *N*-acetyl aspartate (NAA) levels in NFL−/− mice did not differ significantly from NFL+/+ controls (Fig. 2n).

### NFL deletion causes diverse behavioral deficits related to NMDAR hypofunction

**Fig. 3:**
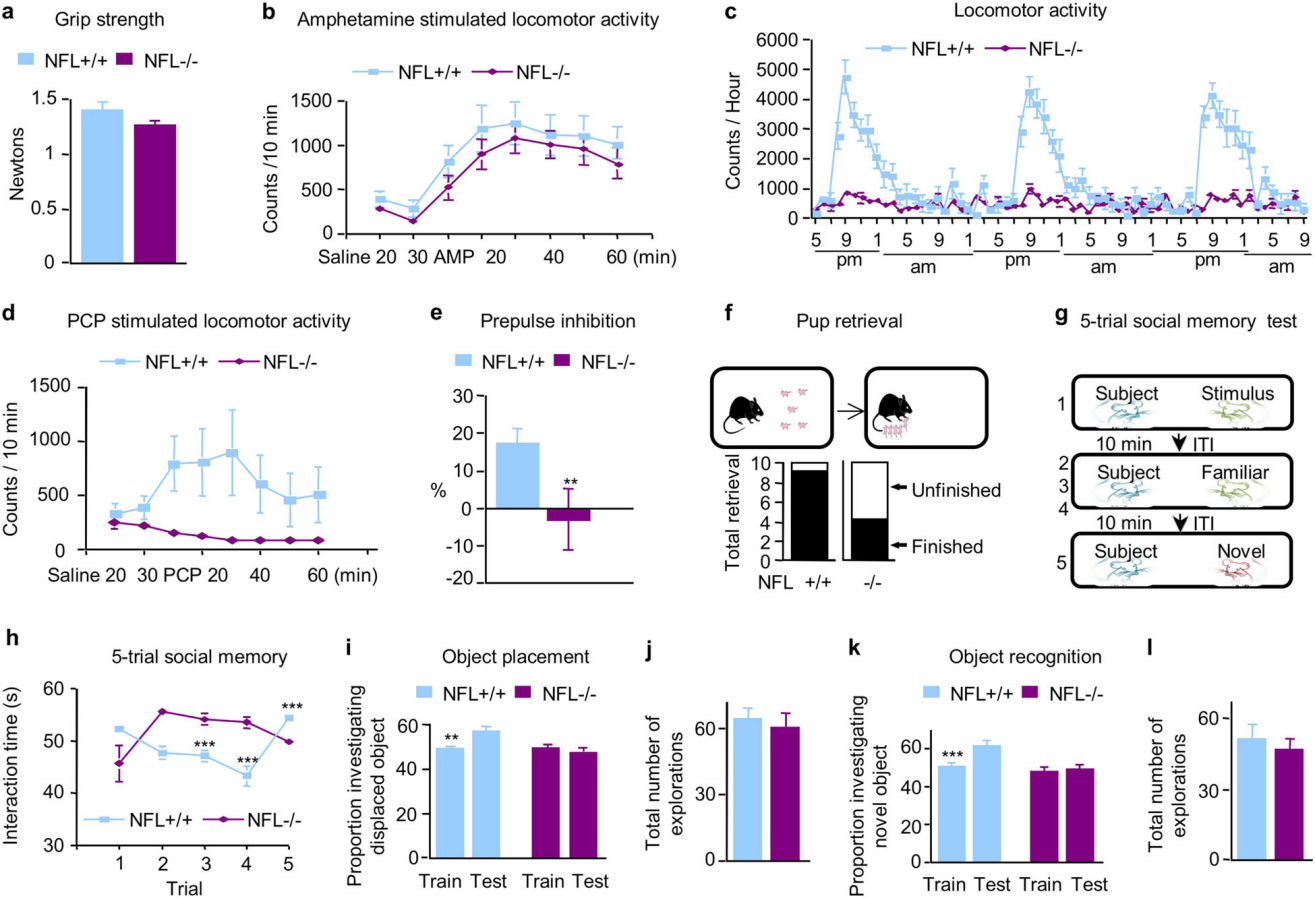
NFL−/− mice have NMDAR-related behavioral deficits and deficits in memory. There were no significant differences in grip strength test (front and back legs) **a** and amphetamine-stimulated locomotor activity between NFL+/+ and NFL−/− mice **b**. **c** Night-time locomotor activity was significantly reduced in NFL−/− mice (*p* < 0.001). All data are presented as mean ± SEM (*n* = 8). **d** NFL−/− mice have reduced stimulant effect of phencyclidine (PCP). Motor stimulant effect of NMDAR antagonist PCP was significantly decreased in NFL−/− mice (*P* < 0.0001). All data are presented as mean ± SEM (*n* = 11–12). **e** NFL−/− mice showed reduced prepulse inhibition (*p* < 0.05). All data are presented as mean ± SEM (*n* = 8). **: *p* < 0.01. **f** Pup retrieval deficit. Schematic representations of the pup retrieval test in a mated pair isolated in an old cage. After cohabitating with their pups as a family unit, the dams were separated from the pups for 10 min and then reunited with five pups. Subsequent pup retrieval behavior was then observed and the number of dams showing retrieval was scored and expressed as a percentage. NFL−/− mice showed a significant deficit in pup retrieval as compared with NFL+/+ and NFL+/− mice (*χ*^2^ test, *n* = 10, *p* < 0.05). **g**, **h** Social interaction deficits in NFL−/− mice based on the five-trial social memory test. NFL+/+ mice displaying normal social recognition showed the standard pattern of high socialization time (trial 1) followed by decreased socialization time (trials 2–4; *P* = 0.0004 at trial 3 and P < 0.0001 at trial 4) and increased socialization time with a novel mouse (trial 5, *P* < 0.0001). NFL−/− mice displaying social recognition defects showed a disrupted pattern of habituation to the same mouse (trials 1–4) and dishabituation to a novel mouse (trial 5, *p* > 0.05). Both female and male NFL−/− mice showed similar direction of change in social memory as compared with NFL+/+ mice. All data are presented as mean ± SEM (*n* = 16). **i**, **j** NFL−/− mice showed a deficit in hippocampus-dependent spatial memory as measured by object placement. NFL−/− mice did not show increased preference for the moved object in a place preference designed to measure hippocampus-dependent spatial memory (*p* > 0.05), whereas NFL+/+ mice did prefer the moved object as measured by a significant increase in the number of investigations of the moved object (*n* = 16, *p* < 0.01). NFL−/− mice showed similar number of investigations for both objects as compared with NFL+/+ mice (*p* > 0.05). All data are presented as mean ± SEM (*n* = 16–17). **k**, **l** NFL−/− mice did not show increased preference for the novel object in a novel preference designed to measure hippocampus-independent visual memory (*p* > 0.05), whereas NFL+/+ mice did prefer the novel object as measured by a significant increase in the number of investigations of the novel object (*p* < 0.01). NFL−/− mice showed similar number of investigations for both objects as compared with NFL+/+ mice (*p* > 0.05). All data are presented as mean ± SEM (*n* = 16–17). **: *p* < 0.01; ***: *p* < 0.001. Both female and male NFL−/− mice showed similar direction of change in both object placement and object recognition as compared with NFL+/+ mice (Supplementary Figure S5)

To rule out the influence of a possible motor deficit on NMDAR-related behavior, we first performed grip strength and amphetamine-stimulated motor activity with NFL−/− mice, which showed no significant impairment of motor capability in NFL−/− mice as compared with NFL+/+ controls (Fig. 3a, b). By contrast, NFL−/− mice displayed markedly reduced night-time locomotor activity at 9 pm as compared with NFL+/+ controls (*P* < 0.001) (Fig. 3c). To implicate NMDAR in this locomotor deficit, we administered phencyclidine (PCP), an NMDAR antagonist that induces motor stimulant effects^29^. The motor stimulant effect of PCP was markedly decreased in NFL−/− mice (*p* < 0.0001) (Fig. 3d), supporting the NMDAR relationship to locomotor deficits. We also found that a 6 dB prepulse of sound induced significantly greater inhibition of the startle reflex in NFL+/+ mice than in NFL−/− animals (Fig. 3e), indicating that NFL deletion leads to a deficit in PPI.

Pup retrieval is a social interaction test and a deficit in pup retrieval in mice has been used as an indicator of abnormal social interaction^30–32^. A pup retrieval test with NFL−/− mice showed that 9 out of 10 NFL+/+ mice finished the pup retrieval task within 10 min, whereas only four out of 10 NFL−/− mice did (Fig. 3f). Statistical analyses established a significant deficit in this innate maternal behavior in NFL−/− mice (*χ*^2^-test, *n* = 10, *p* < 0.05). NFL+/+ control mice displayed normal social memory, as demonstrated by a marked habituation (decreased exploration) during the first four trials and a striking dishabituation (increased exploration) upon the presentation of a novel animal on the 5th trial (Fig. 3g, h). By contrast, NFL−/− mice showed no significant habituation during the four exposures to the stimulus mouse or dishabituation to the novel stimulus mouse indicating a marked deficit in social interaction.

Object-placement task is hippocampus-dependent spatial memory test and glutamatergic transmission abnormalities can cause behavioral deficit in object-placement memory^33,34^. Object placement was assessed in NFL−/− mice with one-trial object-place recognition task in which the mice were allowed to investigate two objects for 5 min during a training session. After 4 h delay, a 5-min test trial was administered in which the mice were allowed to investigate the same two objects, but with one of the objects moved to a novel location. As expected, the NFL+/+ mice investigated the moved object significantly more frequently than the stationary object (*p* < 0.01) (Fig. 3i). By contrast, NFL−/− mice did not show a preference for the moved object, suggesting they have impaired spatial memory of the training configuration. NFL−/− and NFL+/+ mice showed similar numbers of explorations for both objects (Fig. 3j). We also assessed hippocampus-dependent memory with an object recognition task. This test is identical to the object-placement test, except that a novel object is substituted in the same location for one of the training objects during the test trials. Again, NFL+/+ mice showed a significant preference for the novel object, whereas NFL−/− did not (*p* < 0.01) (Fig. 3k). There was no significant difference between the number of explorations for both objects by NFL−/− and NFL+/+ mice (Fig. 3l).

### Partial NFL depletion in NFL+/− mice induces NMDAR-related deficits of synaptic plasticity and behavior

**Fig. 4:**
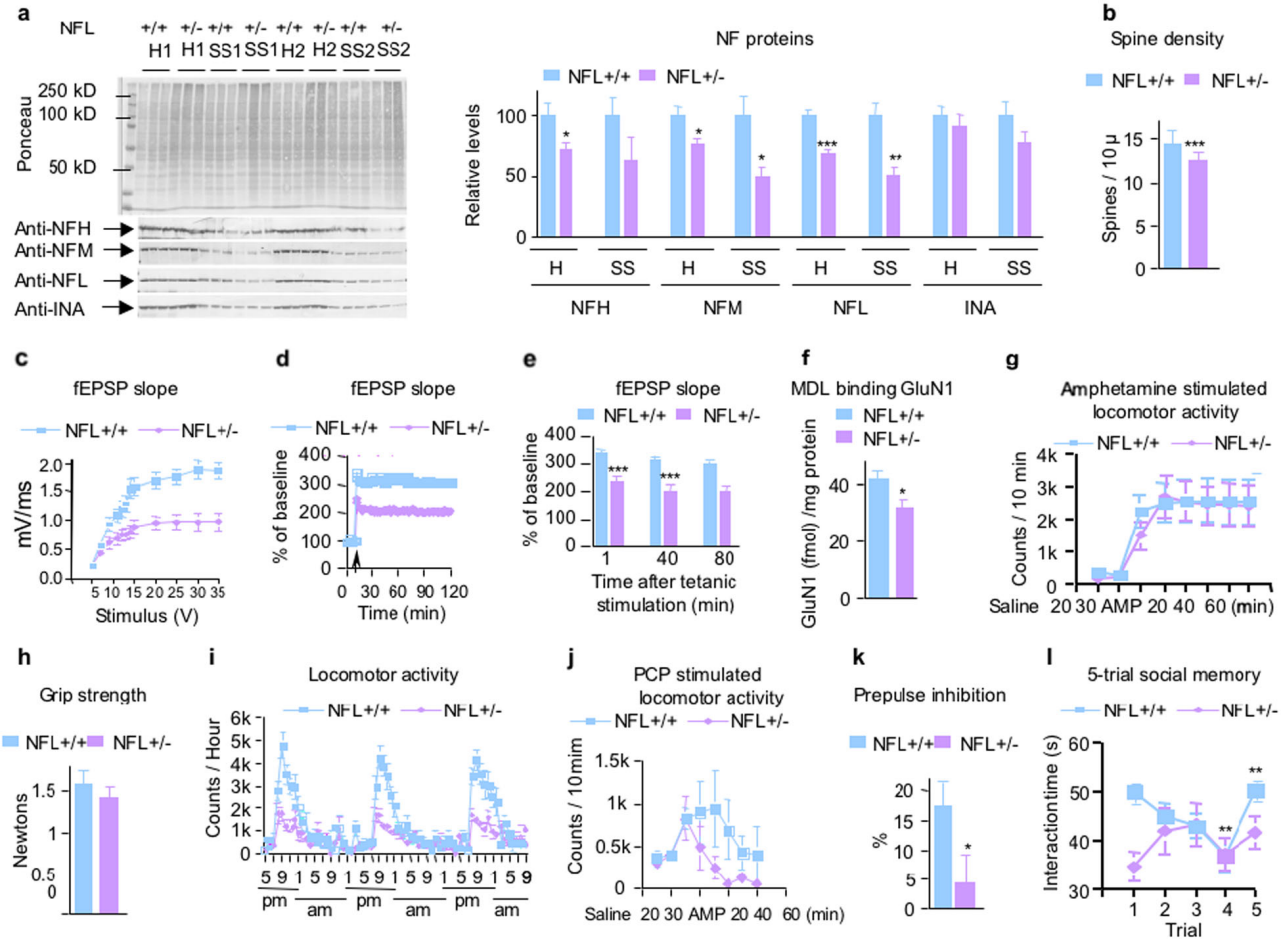
NFL+/− mice showed NMDAR-related partial deficits between NFL+/+ and NFL−/− mice. **a** Analysis of NF protein expression in NFL+/+ and NFL+/− mice. Protein extracts prepared from total hippocampus homogenate and hippocampal synaptosomal fractions were separated on an 8.5% SDS-polyacrylamide gel and transferred to nitrocellulose membrane. Membranes were blotted with different antibodies. Compared with WT levels, NFL, and NFM in NFL+/− mice are lowered 31 and 24%, respectively, in total homogenate (*p* < 0.001) but to 50–51% of WT levels in synaptosomes (*p* < 0.01). NFH and alpha-internexin are lowered 29% (*p* < 0.05) and 9%, respectively, in total homogenate and to 37% and 23%, respectively, in synaptosomes. Data are expressed as Mean ± SEM, *n* = 6. H: homogenate; SS: synaptosomal fractions. **b** Reduced dendritic spine density in the hippocampus of NFL+/− mice. Spine density on CA1 neurons was reduced on dendrites in NFL+/− mice (*P* < 0.01). All data are presented as mean ± SEM (*n* = 8). Impaired CA1 LTP in NFL−/− mice. **c** A summary graph showing the field I/O relationship for NFL+/+ (green) and NFL+/− (red) mice. **d** A time course of the average of the fEPSP slopes from slices obtained from NFL+/+ and NFL+/− mice. **e** A combined plot of the averages of fEPSP slopes at several time points. Each point is the mean ± SEM (*n* = 5 mice per group, 10 slices per group). Two-way ANOVA with Bonferroni’s post hoc test; *p* < 0.001. **f** Reduced level of GluN1 was demonstrated by decreased binding of GluN1 subunit of NMDAR ligand [^3^H]MDL105,519 in the hippocampus of NFL+/− mice. There were no significant differences in amphetamine-stimulated locomotor activity **g** and grip strength test (front and back legs) between NFL++ and NFL+/− mice **h**. **i** Night-time locomotor activity was significantly reduced in NFL+/− mice (*p* < 0.01). Data are presented as mean ± SEM (*n* = 8). **j** Motor stimulant effect of NMDAR antagonist PCP was partially reduced in NFL+/− mice (*p* < 0.05). Data are presented as mean ± SEM (*n* = 8). k NFL+/− mice showed reduced prepulse inhibition (*p* < 0.05). All data are presented as mean ± SEM (*n* = 24–26). **l** Social interaction deficits in NFL+/− mice based on the five-trial social memory test. NFL+/+ mice displaying normal social recognition showed the standard pattern of high socialization time (trial 1) followed by decreased socialization time (trials 2–4; *p* < 0.05 at trial 3 and *p* < 0.01 at trial 4) and increased socialization time with a novel mouse (trial 5, *P* < 0.01). NFL+/− mice displaying social recognition defects showed a disrupted pattern of habituation to the same mouse (trials 1–4) and dishabituation to a novel mouse (trial 5, *p* > 0.05). All data are presented as mean ± SEM (*n* = 8). *: *p* < 0.05; **: *p* < 0.01; ***: *p* < 0.001

To determine the effects of a less-severe loss of NFL as might be associated with disease, we investigated certain of the above studied parameters in NFL+/− as compared with wild-type control mice. Immunoblot analyses of NF subunit protein levels revealed that NFP stoichiometry changed in different ways in hippocampal homogenate and synaptosomes from NFL+/− mice (Fig. 4a). Subunit levels were altered more in synaptosomes than in homogenates. NFL and NFM were reduced to half of the WT level (*p* < 0.01): NFH and alpha-internexin were less affected (63% and 77% of wild-type, respectively) than NFL and NFM. Previous studies also showed that the level of NFL protein decreased by 40% in the brain of NFL+/− mice^35^ and the levels of NFH, NFM, and INA in the cortex vary with age in the absence of NFL^36^. This extent of NF subunit change in synapses was associated, however, with significant synaptic dysfunction.

NFL+/− mice exhibited significantly reduced spine density (*p* < 0.001, *n* = 8) on dendritic arbors of CA1 neurons although spine lengths were unaltered (Fig. 4b). Similar effects of partial deletion of NFL were seen not only on spine density (*p* < 0.01) of dentate gyrus granule cells (Supplementary Figure S6a, b) but also with reduced spine length (*p* < 0.001) (Supplementary Figure S6c), indicating regional difference. Moreover, hippocampal basal synaptic neurotransmission and LTP in the Schaffer collateral pathway of hippocampal slices were significantly impaired in NFL+/− as compared with NFL+/+ mice (*p* < 0.001, *n* = 5) (Fig. 4c–e). GluN1 levels in the hippocampus of NFL+/− mice determined by ligand-binding assay using [^3^H]MDL105,519 were significantly reduced (Fig. 4f). NFL+/− mice showed a significantly diminished night-time locomotor activity at 9 pm as compared with NFL+/+ controls (*p* < 0.001, *n* = 8) (Fig. 4i). The motor stimulant effect of PCP was also significantly decreased in NFL+/− mice (*p* < 0.05, *n* = 9) (Fig. 4j). We also found that a 3 dB prepulse of sound induced a significantly greater inhibition of the startle reflex in NFL+/+ mice than in NFL+/− animals (*n* = 24–26, *p* < 0.05) (Fig. 4k), indicating that NFL partial deletion could lead to deficits in PPI. NFL+/− mice also showed a significant deficit in social interaction (*p* < 0.05, *n* = 8) (Fig. 4l). As shown in Fig. 4g, h, there was no significantly impaired motor capability in NFL+/− mice as compared with NFL+/+ controls as measured by grip strength and amphetamine-stimulated motor activity.

## Discussion

In this report, we demonstrate that NFL is a critical component of synaptic spines and is essential for maintaining spine structural integrity and function. We present multiple lines of evidence showing that NFL modulates NMDAR level and function through a direct in vivo interaction with the GluN1 subunit, which protects against GluN1 ubiquitination and turnover. Finally, we show that even partial reductions in NFL levels, conceivably attainable in some neuropsychiatric states, have substantial synaptic and behavioral effects related to lowered GluN1 levels and diminished NMDAR activity.

NFL deletion is known to decrease axon caliber expansion and dendritic growth of large motoneurons during brain development^26,37^ and NFL’s role in large myelinated peripheral axons has been a dominant focus of most neurofilament investigations. Consistent with these findings, we also found significantly reduced dendrite length, reduced spine density and spine length in dentate gyrus granule cells and reduced spine density and length in CA1 neurons of the hippocampus in NFL−/− mice even though neurofilaments in CNS neurons contribute minimally to axon caliber as demonstrated in our double and triple NF subunit deletion mice^5,6^. Dendritic spine pathology is known to be associated with various neuropsychiatric disorders that are also associated with NF protein alterations^38–40^ and our findings strongly suggest that loss of NFL from synapses may contribute to the spine instability and loss seen in some diseases. Notably, there is reduced dendritic spine density on cerebral cortical pyramidal neurons in schizophrenia^41^. In support, we saw that even a partial reduction in NFL (40%), in the range of losses seen in vulnerable brain regions in schizophrenia, are associated with spine abnormalities.

We further showed that the spine alterations in NFL-deleted mice are accompanied by a marked impairment in basal synaptic transmission and LTP. For the first time, NFL−/− deletion in mice is shown to lower NMDAR–GluN1 protein levels and suppress NMDAR complex function while raising brain glutamate concentrations in hippocampus, presumably as a compensatory response^42–45^. Loss of NFL impaired NMDAR stability, synaptic signaling, and function in mediating behaviors known to be related to activity of this receptor. The selective decrease of GluN1 subunit associated with NFL deletion is consistent with previous evidence that exogenous NFL subunit co-expressed with GluN1 subunit in HEK 293 cells increased GluN1 cell surface expression^12^ and prevented its ubiquitination in vitro^46^. In our study, we demonstrated that K48-linkage-specific ubiquitin signals are significantly increased in GluN1-rich postsynaptic membranes from the hippocampi of NFL−/− mice. This finding and the reduced levels of GluN1 suggest that, when NFL interaction with the receptor is lost, GluN1 degradation by the ubiquitin-proteasome system is increased, consistent with evidence that GluN1 is a substrate of the UPS^46,47^.

Using proton magnetic resonance spectroscopy (MRS), a noninvasive method to measure neurotransmitter concentrations in vivo. we detected elevated glutamate and glutamine concentrations in the hippocampus, of NFL−/− mice as compared with NFL+/+ controls. Although individual 1H-MRS glutamate studies in schizophrenia have produced some inconsistent findings, a recent meta-analysis of proton MRS studies demonstrated schizophrenia is associated with elevations in glutamatergic metabolites across several brain regions^44^. Increased glutamate and glutamine concentrations in the hippocampus of NFL−/− mice could be a result of decreased levels of NMDAR and could reflect the attempt of the system to compensate for postsynaptic glutamatergic hypofunction due to increased GABA disinhibition^45^.

NFL−/− mice display a range of behavioral deficits associated with schizophrenia and in animal models of NMDAR hypofunction^48^ or GluN1 reduction^49,50^, including significant deficits of hippocampus-dependent social memory^34^. Pup retrieval behavior, a measure of hippocampus-dependent nonlearned innate behavior^51,52^ was markedly impaired. Also, object-placement and object-recognition tasks, which are hippocampus-dependent spatial memory tests^34,53^ were deficient in NFL−/− mice. Prepulse inhibition, a characteristic feature of patients with schizophrenia and certain other psychiatric diseases, was also reduced in NFL−/− mice. NFL−/− mice also have a reduction in night-time spontaneous locomotor activity that shows up most clearly at peak time points, suggesting that NFL has effects on locomotor systems that are not clearly a mimic of motor or sleep abnormalities found in schizophrenia^54–56^. Although early studies reported increased locomotor activity in mice expressing 5% of normal levels of GluN1^22^, recent investigation showed that locomotor activity in these mice is comparable to the locomotor activity of wild-type littermates in a familiar environment^57^. In fact, locomotor activity is not directly correlated to the levels of GluN1, as increased locomotor activity has been reported to be associated with both increase^58^ or decrease^59^ of GluN1 levels. PCP is often used to model aspects of schizophrenia, inducing their prominent psychotomimetic effects by blocking neurotransmission at NMDAR-type glutamate receptors and inducing schizophrenia-related behaviors^16,60–64^. The present results showing a lack of response to PCP-induced locomotor activation in the NFL−/− mice further suggest altered NMDAR-mediated mechanisms although one might expect increased sensitivity to PCP-induced locomotor effects in schizophrenic patients.

Our results generally parallel the deficits reported for GluN1 reduction models^49,50^. As NMDAR hypomorph mice show up to 95% reduced expression of GluN1^22,50^, a reduction much greater than what has been shown in postmortem studies of schizophrenia brains^65,66^, heterozygous GluN1−/− mice exhibiting a 30% reduced expression of GluN1 have been proposed to be more disease relevant in schizophrenia studies^50^. A 36% reduction of GluN1 protein was reported in postmortem brain (dorsolateral prefrontal cortex) from people with schizophrenia^66^. Although other studies reported inconsistent findings^67,68^, a recent meta-analysis of five studies of GluN1 protein, consisting of 95 subjects with schizophrenia and 95 controls, indicates a significant decrease in expression of GluN1 protein in schizophrenia subjects relative to controls^68^. Consistent with these findings from human studies, a 30% reduction of GluN1 protein in rat hippocampus induced by GluN1-antisense treatment produced deficits of prepulse inhibition, a well-defined finding in schizophrenia^49^.

**Fig. 5:**
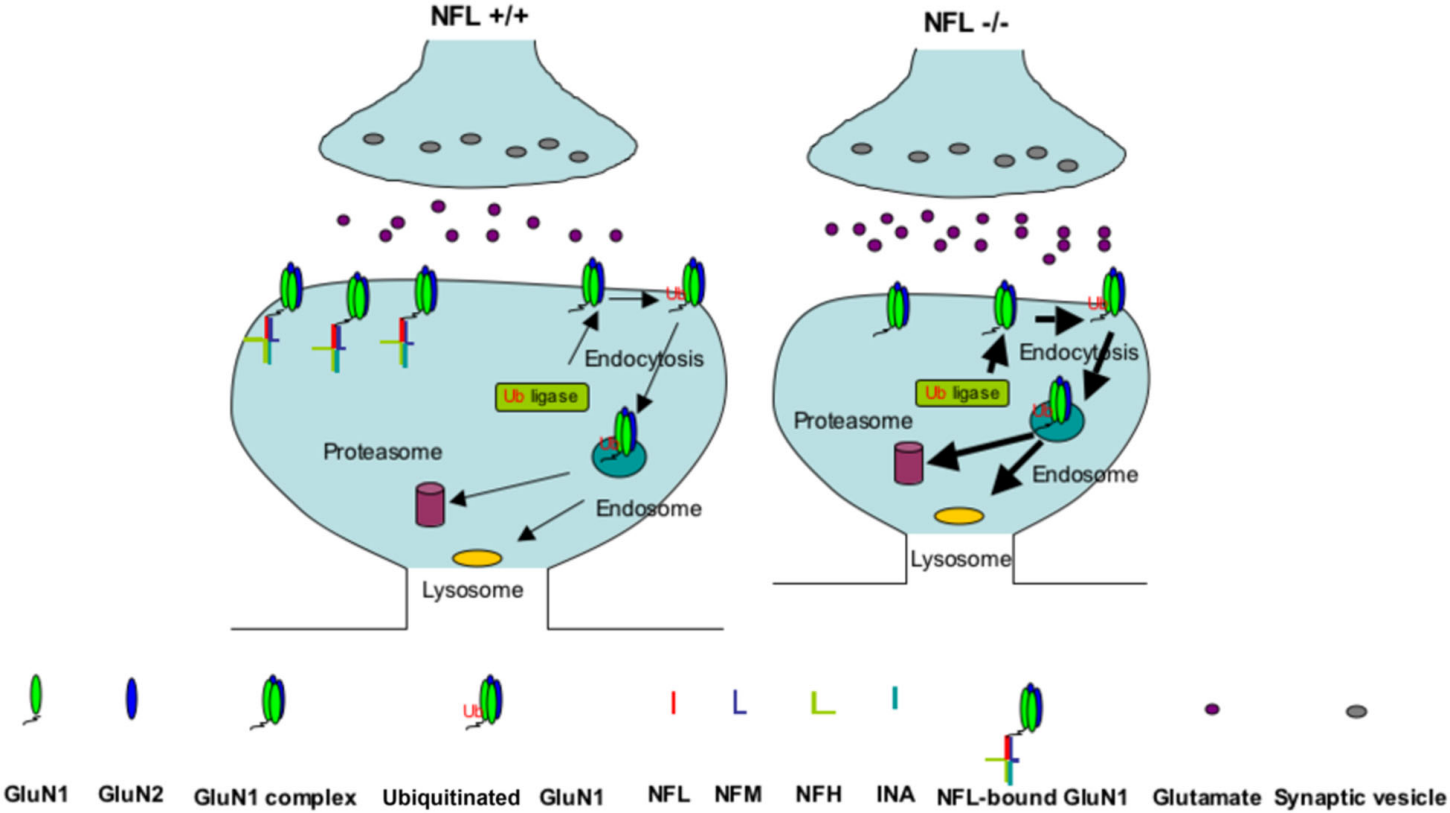
Model of NMDAR binding to NFL. On the basis of collective findings on NF scaffolding functions and our GluN1 data on NFL deletion mice, we propose a model by which NFL acts in synaptic terminals to bind NMDAR on postsynaptic terminals to stabilize the level by protecting against ubiquitination of GluN1. In NFL+/+, GluN1 subunits are physiologically ubiquitinated and degraded as indicated by thin arrows; in NFL−/−, greater ubiquitination and degradation of GluN1 leading to reduced NMDAR function as indicated by thick arrows

Modeling closely the NFL declines reported in affected brain regions in schizophrenia, NFL+/− mice exhibiting a 40% reduction in NFL levels also have significant NMDAR-related deficits. Levels of NFL protein are decreased ~ 50% (statistically significant) by western blot in the dorsolateral prefrontal cortex and ~ 40% (not significant) in the anterior cingulate cortex from patients with schizophrenia^23^. Although 15% increase of NFL transcript was initially reported (these effects were across the collapsed layers and none showed isolated significant changes in a specific isodense band)^23^, a later study by the same group demonstrated ~ 20% (statistically significant) reduction of NFL transcript in the schizophrenic dorsolateral prefrontal cortex (layer V)^69^. Many recent unbiased proteomic studies also demonstrated consistent and significant reduction of NFL subunits in brain regions essential for the cognitive and behavior functions affected in schizophrenia (Supplementary Table S2). The fact that NFL, NFM and NFH genes map to chromosomal regions (8p21, 8p22, and 22q12, respectively) that are strongly implicated in schizophrenia raises the possibility of an involvement of NF proteins in this disease^70,71^. NFL showed consistently reduced expression in three brain regions (anterior cingulate gyrus, motor cortex, and thalamus) following gene expression analysis of postmortem brain tissue of autism patients^72^. NFL also showed genetic association with autism in Caucation families^72^. Our findings are consistent with a model (Fig. 5) in which NFL within a synaptic cytoskeletal lattice binds to the GluN1 receptor and maintains its levels on the postsynaptic surface by preventing its ubiquitination and turnover. Lowered levels of NFL, a substrate for calpains^73^, proteasome^74^, and autophagy^75^ (Rao et al. to be submitted), result in greater ubiquitination and degradation of GluN1 receptors and possibly disruption of key PP1-CamKII interactions with the NMDAR leading to hypofunction of NMDAR signaling. Recent studies of synaptic scaffold proteins suggest that they may form large protein networks and play a major role in synaptic function including the trafficking, anchoring, clustering, and stability of glutamate receptors^76^. Our present results together with previous findings^77–79^ document the likely interactions of NFL, and NF proteins in general, in the scaffold of networked proteins (Supplementary Figure S1) and in the dysfunction of the synaptic scaffold proteins as a molecular basis for severe neuropsychiatric disorders (Supplementary Table S1).

## Electronic supplementary material

Supplemental clean version

Supplemental Figure S1

Supplemental Figure S2

Supplemental Figure S3

Supplemental Figure S4

Supplemental Figure S5

Supplemental Figure S6

Supplemental Table S3
