# Supplementary material for: Neurofilament light interaction with GluN1 modulates neurotransmission and schizophrenia-associated behaviors
Source: Transl Psychiatry. 2018 Aug 24;8:167. doi: 10.1038/s41398-018-0194-7 (PMC6109052; doi:10.1038/s41398-018-0194-7)
Supplement: Supplementary file 1 — Supplemental clean version [file 41398_2018_194_MOESM1_ESM.docx]

**Supplemental Information**

**For**

**“Neurofilament Light Interaction with GluN1 Modulates Neurotransmission and Schizophrenia-Associated Behaviors*”***

**Aidong Yuan^1,7^, Veeranna^1,7^, Henry Sershen^2,7^, Balapal S. Basavarajappa ^3,7, 11, 12^, John F. Smiley^2^, Audrey Hashim^2^, Cynthia Bleiwas^2^, Martin Berg^1^, David N. Guifoyle^4^, Shivakumar Subbanna^3^, Sandipkumar Darji^1^, Asok Kumar^1^, Mala V. Rao^1,7^, Donald A. Wilson^5,9,10^, Jean-Pierre Julien^13^, Daniel C. Javitt^6, 11^** **and Ralph A. Nixon^1,7,8,10^**

^1^Center for Dementia Research, ^2^Neurochemistry Division, ^3^Analytical Psychopharmacology Division, ^4^Center for Biomedical Imaging and Neuromodulation, ^5^Emotional Brain Institute, ^6^Schizophrenia Research, Nathan Kline Institute, Orangeburg, New York 10962

^7^Departments of Psychiatry, ^8^Cell Biology, ^9^Child and Adolescent Psychiatry, and the ^10^Neuroscience Institute, New York University School of Medicine, New York, NY 10016

^11^Department of Psychiatry, College of Physicians & Surgeons, Columbia University, New York, NY 10032

^12^New York State Psychiatric Institute, New York, NY 10032

^13^Centre de Recherche du Centre Hospitalier de l'Université Laval, Département d'anatomie et physiologie de l'Université Laval, 2795 boul. Laurier, Québec G1V 4G2, Canada

**Materials and Methods**

**Generation of Knockout Mice**

Production of NFL -/- mice has been described previously ^1^. NFL -/- mice were compared with wild-type mice of the C57BL/6J strain. Genotyping of the mice was performed to produce NFL -/-, NFL+/- and NFL+/+ mice according to our previous publication. At the beginning of the tests, the mice were from 3 to 5 months of age for most experiments. Mice were single-housed in cages at 23^0^C on a 12-h light-dark cycle and maintained on Lab Chow (Purina Mills, Gray Summit, MO) supplied at libitum and evaluated in separate room. All mice were cared for in accordance with the principles of laboratory animal care and the experimental protocols were approved by NYU/NKI IACUC Committees under the guidelines of the Institutional Animal Care and Use Committee of the United States.

**Golgi staining and quantification of dendritic spine density**

Golgi staining was performed using the FD Rapid GolgiStain kit (FG NeuroTechnologies) as previously described ^2^. Spines were counted on two unobscured apical dendritic branches per neuron, with the average spine density used as the value for that neuron. Spines were counted on three to six neurons per animal. The experimenter was blind to the genotype during tracing.

**^1^H Magnetic Resonance Spectroscopy (MRS)**

All MRS data were acquired on a 7.0 T Agilent (Santa Clara, CA, USA) 40 cm bore system equipped with a gradient coil of 12 cm internal diameter achieving a maximum gradient strength of 600 mT/m and minimum rise time of 200 μs, with customized second and third order shim coils. A Rapid (Rimpar, Germany) volume transmit coil (72mm ID) and a two-channel receive-only surface coil was used for RF transmission and reception, respectively. All animals were anesthetized using an isoflurane vaporizer set at the following percentages: 3% for induction, 2% during pilot scanning and 1.5% during data acquisition. An animal monitoring unit (model 1025, SA Instruments, Stony Brook, NY, USA) was used to record respiration and rectal temperature. Respiration was measured with a pressure transducer placed under the abdomen just below the ribcage. Body temperature was maintained using forced warm air, controlled by a feedback circuit between the heater and thermistor. After induction, the animals were placed on a holder and restrained using a bite bar and ear bars placed half way into the auditory canal. Oxygen was used as the carrier gas and delivered at a low flow rate (≤ 0.5 L/min) to a cone positioned before the bite bar, where gases mixed with air and passed over the rodent’s nose. All animals were maintained at 37.0 ± 0.2 °C. The spectral acquisition consisted of a short echo time Point Resolved Spectroscopy (PRESS) ^3^ sequence with the following parameters: repetition time 4 s, echo time 7.5 ms, number of averages 512 (34 min acquisition), number of points 2048, and bandwidth of acquisition 5 kHz. The shim settings for the selected voxel of interest (VOI) were automatically adjusted using FASTMAP, Automatic Shimming Technique by Mapping Along Projections ^4^. Water suppression was achieved by using Variable Power RF pulses with optimized relaxation delays ^5^. Outer volume suppression was also used. The VOI size was 5 µl (1.0 x 2.0 x 2.5 mm^3^) placed in the right hippocampus. A reference scan with no water suppresion was also acquired. A coronal anatomical pilot scan was used for VOI placement. As a metric of a reliable fit, LCModel outputs the estimated Cramer-Rao lower bounds (CRLB) expressed in percent of the estimated concentration, the lower this number the better the fit. For Glu, NAA and Glu + Gln, the CRLB was < 5% for all animals and < 12% for Gln. All data were processed using the LCModel software developed by Provencher ^6^. This software calculates the best fit to the acquired data of linear combination of model spectra acquired from *in vitro* solutions acquired at the same field strength, sequence, and echo time.

**Long Term Potentiation**

Mice were decapitated following cervical dislocation and hippocampi were quickly dissected and cut into 400µm transverse sections and recorded using standard procedure described earlier ^7^. The hippocampal slices were placed in a recording chamber and perfused with artificial cerebrospinal fluid (ACSF) maintained at 29^0^C with constant bubbling of 95% O_2_ and 5% CO_2_. The ACSF contains 124mM NaCl, 4.4mM KCl, 1mM Na_2_HPO_4_, 25 mM NaHCO_3_, 2 mMCaCl_2_, 2mM MgSO_4_ and 10mM Glucose providing and osmolarity of 290-300. Both the recording and stimulating electrodes were placed in CA1 stratum radiatum to record CA1 field EPSP (fEPSPs). The stimulus voltages were plotted against the slopes of fEPSPs to determine the basal synaptic transmission. A baseline was recorded every minute for 10 minutes at an intensity of the stimulus that evokes 35% of the maximum response. Theta burst stimulation was applied to induce long-term potentiation (LTP; 4 pulses at 100 Hz with burst repeated at 5Hz and each tetani including 3-10 burst trains separated by 15s). The elicited response was recorded for 120 mins as fEPSP slope and expressed as percent baseline.

**Western Blot analyses**

The synaptosomal fractions prepared from hippocampi of mutant and wild-type mice were washed twice with 0.1mM CaCl_2_, using a Beckman Ultra Centrifuge, at 4^0^C (50,000g for 30 min) and then were dissolved in 8M urea mixed with equal volume of buffer 2. The protein concentration was measured with the bicinchonic acid assay (Sigma-Aldrich, St. Louis, MO). One-dimensional SDS-PAGE was performed using slab gels containing 10% polyacrylamide. PAGE-separated proteins were transferred to nitrocellulose membranes overnight using a Genie Blotter (Idea Scientific MN). Blots were probed with NFL (NR4, Sigma-Aldrich), and GluN1 (#4204, Cell Signaling Technology and #05-432, Millipore), NR2B (ThermoFisher) and GAPDH (Santa Cruz), Na, K-ATPase (# 3010, Cell Signaling Technology), K48- and K63-linkage specific polyubiquitin (#5621 and #8081, Cell Signaling Technology) antibodies and developed using peroxidase conjugated secondary antibody and an ECL kit (GE) or DAB kit (ABC).

**Immunoelectron microscopy**

NFL-/- and NFL+/+ mice, anesthetized with isoflurane, were perfused intracardially with 4% paraformaldehyde in 0.1M phosphate buffer, pH 7.4. NFL was detected using a mouse monoclonal antibody (NR4, Sigma), followed by 15-nm gold conjugated goat anti-mouse. NFH was detected using polyclonal antibody (Sigma), followed by 10-nm gold conjugated goat anti-rabbit. GluN1 was detected using polyclonal antibody (#4204, Cell Signaling Technology and #05-432, Millipore), followed by 10-nm gold conjugated goat anti-rabbit. The grids were photographed on a JEOL 100 cx electron microscope operated at 80 kV.

**Immunoprecipitation of NFL and GluN1**

Immunoprecipitation of NFL and GluN1 from hippocampal synaptosomal preparations was performed with specific antibodies according the procedures described. Antibodies used are anti-NFL (NR4, Sigma-Aldrich), anti-GluN1 NT (#07-362, Upstate cell signalling solutions) and anti-GluN1 CT (#05-432, Millipore). The immunoprecipitates subsequently were analyzed by SDS-PAGE and Western blot analysis.

**Radioligand binding assay**

[^3^H]MDL105,519 (Perkin Elmer NET 1171) binding assays were carried out using a polyethyleimine filtration assay with 100 µM glycine for the determination of non-specific binding according to published procedure ^8^.

**Locomotor activity**

A group of 16 NFL+/+ (6 males, 10 females), 17 NFL-/- (8 males, 9 females) and 22 NFL+/- (11 males, 15 females) littermate mice between 3 – 4 months old were used sequentially for locomotor activity and grip strength test. The NFL+/+ and NFL-/- mice were further used for object placement, object recognition and 5-trial social memory test*.* New groups of age-and sex-matched NFL+/+ and NFL-/- or NFL+/+ and NFL+/- mice were used sequencially for PCP-and amphetamine -treated tests. Mice were housed individually in standard mouse cages (7 x 12 x 5 inches). The cages were placed in the activity monitors (Opto Varimex- 3 photocell activity monitors , inter-beam distance 1 inch apart, Columbus Instruments, Columbus, Ohio). For overnight activity, activity was measured between 4:00 pm to 8:00 am. Lights were off at 6:00 pm and on at 6:00 am. Locomotor activity was calculated based on total ambulatory counts (consecutive beams broken during ambulation, single beams broken repeatedly are not counted). Data were expressed as ambulatory beam breaks over the 16-hour period (in 1 hour segments). With drug treatments, the cage is placed in the activity monitor and activity measured starting after drug injection(s) over a 60-90 min period (in 10 min segments). Phencyclidine-HCl (NIH)(PCP) and amphetamine sulfate (Sigma-Aldrich, St. Louis, MO) were given at 5 mg/kg, ip.

**Prepulse inhibition**

A group of eight 50-day old NFL-/- and eight 50-day old NFL+/+ mice were used for this test. Mice were restrained in a tube on a platform situated in a chamber (Kinder Scientific) in which the involuntary startle response is measured by an accelerometer underneath the platform. The equipment was operated with software to expose the mice to the following trials: no startle noise, just the startle noise (100 db), or a prepulse (6 db overbackground) before startle noise. Background noise was 65 db. Prepulse inhibition was calculated as follows: 100% X ((startle.pulse)-(startle.prepulse))/(startle.pulse).

**5-trial social memory**

Mutant mice were compared with wild-type control mice of the same background strain (C57BL6). The test was carried out according to our published procedures ^9^. These mice were 4-6 months old at the time of the test and individually housed for 7 days to permit establishment of a home-cage territory. All test trials were videotaped and subsequently analyzed for investigation time. The first trial began by introducing a stimulus female mouse (never-before-met) into the home cage of a mouse for a 1-min interaction. At the end of the 1-min trial, the stimulus animal was removed and returned to an individual holding cage. For the second trial, after a 10-min inter-trial interval, the same stimulus female was introduced to the mouse for 1-min and later removed to the individual holding cage. For the third and fourth trials, steps were repeated as the second trial. In the fifth “dishabituation” trial, a different stimulus female mouse was introduced to the same mouse for 1-min and later removed to the individual holding cage.

**Pup Retrieval**

New groups of virgin female and male mutant and wild type control mice (10 female and 10 male animals of each genotype) are used soley for this behavior study. These mice are paired and continuously housed together in a standard mouse cage. The cages are left undisturbed during the 0-2 days after the first litter produced by each pair. The pups of each litter and sires are removed during 3-5 days after birth and placed in a separate holding cage for 10 min while dams are left in the original cages. Five pups from the litter are then returned to the original cage but placed in a remote area away from the nest. When dams are re-united with their pups, they will pick up a mouse pup using their mouths and physically move it from outside to inside the nest. The test ends when all pups have been retrieved or if all pups have not been returned to the nest site within 10 min ^10^.

**Object placement (OP) behavior**

This procedure takes three days and tests the spatial memory in the mice. The OP task is a hippocampal dependent spatial memory test designed to measure memory of object location in an open field following a predetermined inter-trial interval. The test was performed according to our previous publication ^11^. Briefly, mice were acclimated to an open Plexiglas arena (20 cm x 40 cm x 20 cm) lined with fresh corn cob bedding twice a day for 5 min each with inter-trial intervals of 5 min on day 1, and 4 h on day 2. On the third day mice were placed in the same arena with two identical objects (A and B; amber glass vials, 2 cm diameter, 5 cm tall) placed equidistant to adjacent corners of the arena. Four hours after training, one object (B) was moved longitudinally to a position diagonal from the object retaining its original position. Behavior on the training and testing days was videotaped for off-line analyses. Investigations were defined as head movements in the direction of the object in which the tip of the nose was within 2 cm of the object and was quantified over a 5-min time period. The locations of the objects will be equidistant from the corners in a diagonal fashion and positioning counterbalanced. The preference for the moved object was measured by calculating the number of investigations of each object.

**Object Recognition(OR) behavior**

Two weeks after object placement test, mice were subject to object recognition test. This test was performed similar to the above object placement test except that amber glass vials were changed to glass beakers (2.5 cm diameter, 3.3 cm tall) and one of the beakers was replaced with a novel object (binder clip, 2 cm x 3 cm) in the same location instead of one of the beakers moved to a different location.

**Grip strength test**

A grip strength meter (Bioseb) was used to assess front and back leg grip strength. Mice were lifted and held by their tail so that their four paws could grasp a wire grid. The mice were then gently pulled backwards by the tail until they released the grid. The peak force applied by the four paws of the mouse was recorded in Newton (N). Each mouse was tested three times, and the greatest measured value was used for statistical analysis.

**Animal genders in experiments**

Groups of age-and sex-matched knockout and control mice were used in all experiments. For NFL-/- experiments, twelve female NFL-/- and 12 female NFL+/+ mice (hippocampi from two mice pooled) were used in the Western blot analysis of NF subunits proteins. Eight female NFL-/- and 8 female NFL+/+ mice were used in the analysis of dendritic spines. Seven male NFL-/- and 7 male NFL+/+ mice were used in the LTP analysis. Four male NFL-/- and 4 male NFL+/+ mice were used in the IEM analysis of GluN1. Eight female NFL-/- and 8 female NFL+/+ mice were used in the radioligand binding assay. Eight female NFL-/- and 8 female NFL+/+ mice were used in the Western blot analysis of GluN1 protein. Twelve female NFL-/- and 8 female NFL+/+ mice were used in the Western blot analysis of K48-linkage specific ubiquitin signals. Sixteen NFL-/- (8 females and 8 males) and sixteen NFL+/+ mice (10 females and 6 males) were used in ^1^H Magnetic Resonance Spectroscopy. Eight female NFL-/- and 8 female NFL+/+ mice were used in grip strength test. Twelve male NFL-/- and 11 male NFL+/+ mice were used for PCP-and amphetamine -treated tests. Eight male NFL-/- and 8 male NFL+/+ mice were used for the test of night-time locomotor activity. Eight female NFL-/- and 8 female NFL+/+ mice were used for the test of prepulse inhibition. Ten female NFL-/- and ten female NFL+/+ mice were used for the test of pup retrieval. Sixteen NFL-/- (8 females and 8 males) and sixteen NFL+/+ mice (10 females and 6 males) were used in 5-trial social memory test. Seventeen NFL-/- (9 females and 8 males) and sixteen NFL+/+ mice (10 females and 6 males) were used in object placement and object recognition tests.

For NFL+/- experiments, twelve female NFL+/- and 12 female NFL+/+ mice (hippocampi from two mice pooled) were used in the Western blot analysis of NF subunits proteins. Eight female NFL+/- and 8 female NFL+/+ mice were used in the analysis of dendritic spines. Five male NFL+/- and 5 male NFL+/+ mice were used in the LTP analysis. Eight female NFL+/- and 8 female NFL+/+ mice were used in the radioligand binding assay. Eight female NFL+/- and 8 female NFL+/+ mice were used for PCP-and amphetamine -treated tests. Eight female NFL+/- and 8 female NFL+/+ mice were used in grip strength test. Eight male NFL+/- and 8 male NFL+/+ mice were used for the test of night-time locomotor activity. Twenty-four female NFL+/- and 26 female NFL+/+ mice were used for the test of prepulse inhibition. Eight female NFL+/- and 8 female NFL+/+ mice were used in 5-trial social memory test.

**Statistical analysis**

Sample sizes were chosen according to the standard practice in the field ^9^. Significance was determined using unpaired two-tailed Student’s *t* test, the Mann-Whitney test or Two-way ANOVA with Bonferroni’s *post hoc* test. The variance is similar between the groups that are statistically compared.

**Supplemental Figure Legends**

**Supplemental Figure S1. Protein sub-network of the interactive partners of NEFL and its interactors in dendritic spines**. Only major families of synaptic scaffold or spinosketal proteins (gene names used) are shown. NEFL and its direct interactions are shown in red. The other 3 NF subunits NEFM, NEFH, INA and their direct interactions are shown in yellow. All other synaptic scaffolds are shown in white and black. All of the depicted proteins interacting with the NF proteins are known to be altered in schizophrenia and other neuropsychiatric diseases (See **Supplemental** **Table S1**).

**Supplemental Figure S2. Reduced GluN1 expression in IHL-TKO mice**. In the absence of INA, NFH and NFL, Western blot with anti-GluN1 antibody showed a reduced level of synaptosomal GluN1 from hippocampi whereas the level of GluN2B subunit was not significantly affected. *: p<0.05.

**Supplemental Figure S3. Female and male mice showed similar direction of change in glutamate and glutamine levels**. ^1^H MRS glutamate and glutamine concentration in female NFL-/- versus female NFL+/+ mice in the hippocampus showed a significant increase (**a**-**d**) (Data are presented as mean ± SEM, n=8-10). ^1^H MRS glutamate and glutamine concentration in male NFL-/- versus male NFL+/+ mice in the hippocampus also showed a significant increase (**e**-**h**) (Data are presented as mean ± SEM, n=6-8). *: p<0.05; **: p<0.01.

**Supplemental Figure S4. No significant differences of glutamate levels between female and male mice**. ^1^H MRS glutamate concentration in female NFL+/+ versus male NFL+/+ mice in the hippocampus showed no significant difference (**a**) (Data are presented as mean ± SEM, n=8). ^1^H MRS glutamate concentration in female NFL-/- versus male NFL-/-mice in the hippocampus also showed no significant difference (**b**) (Data are presented as mean ± SEM, n=6-10).

**Supplemental Figure S5. Female (a, c) and male (b, d) mice showed similar direction of change in object placement and recognition tests**. NFL-/- mice did not show increased preference for the novel object in a novel preference designed to measure hippocampus-independent visual memory (p>0.05), whereas NFL+/+ mice did prefer the novel object as measured by a significant increase in the number of investigations of the novel object. Data are presented as mean ± SEM (n=6-10). *: p<0.05; **: p<0.01; ***: p<0.001.

**Supplemental Figure S6.** Reduction of dendritic spine density (a, b) and spine length (c) in dentate gyrus granule cells of NFL+/- mice. Quartile 2, p <0.05; Quartile 3, p <0.01; Quartile 4, p <0.05. *: p<0.05; **: p<0.01

**Supplemental Table S1.** Mutations in the genes encoding the synaptic scaffolding proteins and associations with neuropsychiatric diseases.

**Supplemental Table S2.** Neurofilament light subunit protein expression in schizophrenia.

**Supplemental Table S3.** Reduced path length of dendrites in dentate gyrus granule cells of NFL-/- mice.

**References for Supplemental Materials and Methods**

1. Balu DT, Li Y, Puhl MD, Benneyworth MA, Basu AC, Takagi S*, et al*. Multiple risk pathways for schizophrenia converge in serine racemase knockout mice, a mouse model of NMDA receptor hypofunction. *Proceedings of the National Academy of Sciences of the United States of America* 2013; **110**(26)**:** E2400-2409.

2. Bottomley PA. Spatial localization in NMR spectroscopy in vivo. *Ann N Y Acad Sci* 1987; **508:** 333-348.

3. Gruetter R. Automatic, localized in vivo adjustment of all first- and second-order shim coils. *Magn Reson Med* 1993; **29**(6)**:** 804-811.

4. Tkac I, Starcuk Z, Choi IY, Gruetter R. In vivo 1H NMR spectroscopy of rat brain at 1 ms echo time. *Magn Reson Med* 1999; **41**(4)**:** 649-656.

5. Provencher SW. Estimation of metabolite concentrations from localized in vivo proton NMR spectra. *Magn Reson Med* 1993; **30**(6)**:** 672-679.

6. Sgambato V, Vanhoutte P, Pages C, Rogard M, Hipskind R, Besson MJ*, et al*. In vivo expression and regulation of Elk-1, a target of the extracellular-regulated kinase signaling pathway, in the adult rat brain. *The Journal of neuroscience : the official journal of the Society for Neuroscience* 1998; **18**(1)**:** 214-226.

7. Chazot PL, Reiss C, Chopra B, Stephenson FA. [3H]MDL 105,519 binds with equal high affinity to both assembled and unassembled NR1 subunits of the NMDA receptor. *Eur J Pharmacol* 1998; **353**(1)**:** 137-140.

8. Yuan A, Sershen H, Veeranna, Basavarajappa BS, Kumar A, Hashim A*, et al*. Neurofilament subunits are integral components of synapses and modulate neurotransmission and behavior in vivo. *Molecular psychiatry* 2015; **20**(8)**:** 986-994.

9. Liu HX, Lopatina O, Higashida C, Fujimoto H, Akther S, Inzhutova A*, et al*. Displays of paternal mouse pup retrieval following communicative interaction with maternal mates. *Nat Commun* 2013; **4:** 1346.

10. Kaur G, Sharma A, Xu W, Gerum S, Alldred MJ, Subbanna S*, et al*. Glutamatergic transmission aberration: a major cause of behavioral deficits in a murine model of Down's syndrome. *The Journal of neuroscience : the official journal of the Society for Neuroscience* 2014; **34**(15)**:** 5099-5106.

**References for Supplemental Figure S1**

1. Zhu Q, Couillard-Despres S, Julien JP. Delayed maturation of regenerating myelinated axons in mice lacking neurofilaments. *Experimental neurology* 1997; **148**(1)**:** 299-316.

2. Balu DT, Li Y, Puhl MD, Benneyworth MA, Basu AC, Takagi S*, et al*. Multiple risk pathways for schizophrenia converge in serine racemase knockout mice, a mouse model of NMDA receptor hypofunction. *Proceedings of the National Academy of Sciences of the United States of America* 2013; **110**(26)**:** E2400-2409.

3. Bottomley PA. Spatial localization in NMR spectroscopy in vivo. *Ann N Y Acad Sci* 1987; **508:** 333-348.

4. Gruetter R. Automatic, localized in vivo adjustment of all first- and second-order shim coils. *Magn Reson Med* 1993; **29**(6)**:** 804-811.

5. Tkac I, Starcuk Z, Choi IY, Gruetter R. In vivo 1H NMR spectroscopy of rat brain at 1 ms echo time. *Magn Reson Med* 1999; **41**(4)**:** 649-656.

6. Provencher SW. Estimation of metabolite concentrations from localized in vivo proton NMR spectra. *Magn Reson Med* 1993; **30**(6)**:** 672-679.

7. Sgambato V, Vanhoutte P, Pages C, Rogard M, Hipskind R, Besson MJ*, et al*. In vivo expression and regulation of Elk-1, a target of the extracellular-regulated kinase signaling pathway, in the adult rat brain. *The Journal of neuroscience : the official journal of the Society for Neuroscience* 1998; **18**(1)**:** 214-226.

8. Chazot PL, Reiss C, Chopra B, Stephenson FA. [3H]MDL 105,519 binds with equal high affinity to both assembled and unassembled NR1 subunits of the NMDA receptor. *Eur J Pharmacol* 1998; **353**(1)**:** 137-140.

9. Yuan A, Sershen H, Veeranna, Basavarajappa BS, Kumar A, Hashim A*, et al*. Neurofilament subunits are integral components of synapses and modulate neurotransmission and behavior in vivo. *Molecular psychiatry* 2015; **20**(8)**:** 986-994.

10. Liu HX, Lopatina O, Higashida C, Fujimoto H, Akther S, Inzhutova A*, et al*. Displays of paternal mouse pup retrieval following communicative interaction with maternal mates. *Nat Commun* 2013; **4:** 1346.

11. Kaur G, Sharma A, Xu W, Gerum S, Alldred MJ, Subbanna S*, et al*. Glutamatergic transmission aberration: a major cause of behavioral deficits in a murine model of Down's syndrome. *The Journal of neuroscience : the official journal of the Society for Neuroscience* 2014; **34**(15)**:** 5099-5106.

**Yuan et al. Supplemental Table S1: Mutations in the Genes Encoding the Synaptic Scaffolding Proteins and Neuropsychiatric Diseases**

| **Gene Symbol** | **Mutations** | **Protein Levels** | **Model System** | **Diseases** | **References** |
| --- | --- | --- | --- | --- | --- |
| DLG3 | Frameshift | Decrease | Human | Mental retardation | 1 |
| DLG4 | Polymorphism | N.D. | Human | Schizophrenia  Autism spectrum disorders  Williams syndrome | 2, 3, 4 |
| DLG4 | Null | 100% decrease | Mouse | Impaired learning  Autism spectrum disorders  Williams syndrome | 3, 5 |
| DLGAP2 | Copy number variations | N.D. | Human | Autism spectrum disorders | 6 |
| DLGAP3 | Missense | N.D. | Human | Trichotillomania  Obsessive-compulsive disorder | 7 |
| DLGAP3 | Null | 100% decrease | Mouse | Obsessive-compulsive disorder | 8 |
| SHANK2 | Copy number variations  Nonsense | N.D. | Human | Autism spectrum disorders  Mental retardation | 6, 9 |
| SHANK3 | Missense  Frameshift  Deletion | N.D. | Human | Autism spectrum disorders | 10, 11, 12, 13, 14 |
| SHANK3 | Null | Significant decrease | Mouse | Impaired social interaction | 15 |
| SHANK3B | Null | 100% decrease | Mouse | Autistic-like | 16 |
| HOMER1 | Null | 100% decrease | Mouse | Schizophrenia-like | 17 |
| HOMER2 | Null | 100% decrease | Mouse | Addiction | 18 |
| ANKS1B | Polymorphism | N.D. | Human | Schizophrenia | 19, 20 |
| ANKS1B | Null | 100% decrease | Mouse | Schizophrenia-like | 21 |
| SYNGAP1 | Frameshift  Copy number variations | N.D. | Human | Autosomal nonsyndromic mental retardation  Autism spectrum disorders | 6, 22 |
| SYNGAP1 | Null | 50% decrease | mouse | Schizophrenia-like | 23 |
| Alpha-CaMKII | null | 50% Decrease | Mouse | Schizophrenia-like | 24 |
| PPP1R9B | Duplication | N.D. | Human | Cognitive impairment | 25 |
| PPP1R9B | Null | 100% decrease | Mouse | Impaired learning | 26 |
| IQSEC3 | Missense | N.D. | Human | X-linked intellectual disability | 27 |
| TANC1 | Null | 100% decrease | Mouse | Impaired memory | 28 |
| SRCIN1 | Null | 100% decrease | Mouse | Impaired memory | 29 |
| DISC1 | Frameshift | Decrease | Human | Schizophrenia | 30 |
| LRRC7 | Null | 100% decrease | mouse | Schizophrenia-like  Autism-like | 31 |
| YWHAZ | Null | 100% decrease | mouse | Schizophrenia-like | 32 |
| CTNNB1 | Missense | N.D. | Human | Schizophrenia | 33 |
| CTNND2 | Missense | N.D. | Human | Schizophrenia | 34 |
| NLGN3 | Missense | N.D. | Human | Autism spectrum disorders | 35 |
| NLGN4 | Frameshift | Decrease | Human | Autism spectrum disorders | 35 |
| CACNG2 | Missense | N.D. | Human | Mental retardation, autosomal dominant 10 | 36 |
| SPTBN2 | Missense | N.D. | Human | Spinocerebellar ataxia 5 | 37 |
| ATCB | Missense | N.D. | Human | Baraitser-Winter syndrome (mental retardation) | 38 |
| TUBB | Missense | N.D. | Human | Circumferential skin creases Kunze type (intellectual disability) | 39 |
| MAPT | Missense | N.D. | Human | FTDP-17 | 40 |

**References for Supplemental Table S1**

1. Tarpey P, Parnau J, Blow M, Woffendin H, Bignell G, Cox C*, et al*. Mutations in the DLG3 gene cause nonsyndromic X-linked mental retardation. *Am J Hum Genet* 2004; **75**(2)**:** 318-324.

2. Chen J, Nakata T, Zhang Z, Hirokawa N. The C-terminal tail domain of neurofilament protein-H (NF-H) forms the crossbridges and regulates neurofilament bundle formation. *J Cell Sci* 2000; **113 Pt 21:** 3861-3869.

3. Feyder M, Karlsson RM, Mathur P, Lyman M, Bock R, Momenan R*, et al*. Association of mouse Dlg4 (PSD-95) gene deletion and human DLG4 gene variation with phenotypes relevant to autism spectrum disorders and Williams' syndrome. *Am J Psychiatry* 2010; **167**(12)**:** 1508-1517.

4. Wang J, Li L, Shao SS, He Z, Chen YL, Kong R*, et al*. Association analysis of genetic variant of rs13331 in PSD95 gene with autism spectrum disorders: A case-control study in a Chinese population. *J Huazhong Univ Sci Technolog Med Sci* 2016; **36**(2)**:** 285-288.

5. Migaud M, Charlesworth P, Dempster M, Webster LC, Watabe AM, Makhinson M*, et al*. Enhanced long-term potentiation and impaired learning in mice with mutant postsynaptic density-95 protein. *Nature* 1998; **396**(6710)**:** 433-439.

6. Pinto D, Pagnamenta AT, Klei L, Anney R, Merico D, Regan R*, et al*. Functional impact of global rare copy number variation in autism spectrum disorders. *Nature* 2010; **466**(7304)**:** 368-372.

7. Zuchner S, Wendland JR, Ashley-Koch AE, Collins AL, Tran-Viet KN, Quinn K*, et al*. Multiple rare SAPAP3 missense variants in trichotillomania and OCD. *Mol Psychiatry* 2009; **14**(1)**:** 6-9.

8. Welch JM, Lu J, Rodriguiz RM, Trotta NC, Peca J, Ding JD*, et al*. Cortico-striatal synaptic defects and OCD-like behaviours in Sapap3-mutant mice. *Nature* 2007; **448**(7156)**:** 894-900.

9. Berkel S, Marshall CR, Weiss B, Howe J, Roeth R, Moog U*, et al*. Mutations in the SHANK2 synaptic scaffolding gene in autism spectrum disorder and mental retardation. *Nat Genet* 2010; **42**(6)**:** 489-491.

10. Bonaglia MC, Giorda R, Borgatti R, Felisari G, Gagliardi C, Selicorni A*, et al*. Disruption of the ProSAP2 gene in a t(12;22)(q24.1;q13.3) is associated with the 22q13.3 deletion syndrome. *Am J Hum Genet* 2001; **69**(2)**:** 261-268.

11. Durand CM, Betancur C, Boeckers TM, Bockmann J, Chaste P, Fauchereau F*, et al*. Mutations in the gene encoding the synaptic scaffolding protein SHANK3 are associated with autism spectrum disorders. *Nat Genet* 2007; **39**(1)**:** 25-27.

12. Moessner R, Marshall CR, Sutcliffe JS, Skaug J, Pinto D, Vincent J*, et al*. Contribution of SHANK3 mutations to autism spectrum disorder. *Am J Hum Genet* 2007; **81**(6)**:** 1289-1297.

13. Gauthier J, Spiegelman D, Piton A, Lafreniere RG, Laurent S, St-Onge J*, et al*. Novel de novo SHANK3 mutation in autistic patients. *Am J Med Genet B Neuropsychiatr Genet* 2009; **150B**(3)**:** 421-424.

14. Betancur C, Buxbaum JD. SHANK3 haploinsufficiency: a "common" but underdiagnosed highly penetrant monogenic cause of autism spectrum disorders. *Mol Autism* 2013; **4**(1)**:** 17.

15. Bozdagi O, Sakurai T, Papapetrou D, Wang X, Dickstein DL, Takahashi N*, et al*. Haploinsufficiency of the autism-associated Shank3 gene leads to deficits in synaptic function, social interaction, and social communication. *Mol Autism* 2010; **1**(1)**:** 15.

16. Peca J, Feliciano C, Ting JT, Wang W, Wells MF, Venkatraman TN*, et al*. Shank3 mutant mice display autistic-like behaviours and striatal dysfunction. *Nature* 2011; **472**(7344)**:** 437-442.

17. Szumlinski KK, Lominac KD, Kleschen MJ, Oleson EB, Dehoff MH, Schwarz MK*, et al*. Behavioral and neurochemical phenotyping of Homer1 mutant mice: possible relevance to schizophrenia. *Genes Brain Behav* 2005; **4**(5)**:** 273-288.

18. Szumlinski KK, Dehoff MH, Kang SH, Frys KA, Lominac KD, Klugmann M*, et al*. Homer proteins regulate sensitivity to cocaine. *Neuron* 2004; **43**(3)**:** 401-413.

19. McClay JL, Adkins DE, Aberg K, Stroup S, Perkins DO, Vladimirov VI*, et al*. Genome-wide pharmacogenomic analysis of response to treatment with antipsychotics. *Mol Psychiatry* 2011; **16**(1)**:** 76-85.

20. Kang SG, Chee IS, Lee K, Lee J. rs7968606 polymorphism of ANKS1B is associated with improvement in the PANSS general score of schizophrenia caused by amisulpride. *Hum Psychopharmacol* 2017; **32**(2).

21. Enga RM, Rice AC, Weller P, Subler MA, Lee D, Hall CP*, et al*. Initial characterization of behavior and ketamine response in a mouse knockout of the post-synaptic effector gene Anks1b. *Neurosci Lett* 2017; **641:** 26-32.

22. Hamdan FF, Gauthier J, Spiegelman D, Noreau A, Yang Y, Pellerin S*, et al*. Mutations in SYNGAP1 in autosomal nonsyndromic mental retardation. *N Engl J Med* 2009; **360**(6)**:** 599-605.

23. Guo X, Hamilton PJ, Reish NJ, Sweatt JD, Miller CA, Rumbaugh G. Reduced expression of the NMDA receptor-interacting protein SynGAP causes behavioral abnormalities that model symptoms of Schizophrenia. *Neuropsychopharmacology* 2009; **34**(7)**:** 1659-1672.

24. Yamasaki N, Maekawa M, Kobayashi K, Kajii Y, Maeda J, Soma M*, et al*. Alpha-CaMKII deficiency causes immature dentate gyrus, a novel candidate endophenotype of psychiatric disorders. *Mol Brain* 2008; **1:** 6.

25. Zahir FR, Langlois S, Gall K, Eydoux P, Marra MA, Friedman JM. A novel de novo 1.1 Mb duplication of 17q21.33 associated with cognitive impairment and other anomalies. *Am J Med Genet A* 2009; **149A**(6)**:** 1257-1262.

26. Stafstrom-Davis CA, Ouimet CC, Feng J, Allen PB, Greengard P, Houpt TA. Impaired conditioned taste aversion learning in spinophilin knockout mice. *Learn Mem* 2001; **8**(5)**:** 272-278.

27. Kalscheuer VM, James VM, Himelright ML, Long P, Oegema R, Jensen C*, et al*. Novel Missense Mutation A789V in IQSEC2 Underlies X-Linked Intellectual Disability in the MRX78 Family. *Front Mol Neurosci* 2015; **8:** 85.

28. Han S, Nam J, Li Y, Kim S, Cho SH, Cho YS*, et al*. Regulation of dendritic spines, spatial memory, and embryonic development by the TANC family of PSD-95-interacting proteins. *J Neurosci* 2010; **30**(45)**:** 15102-15112.

29. Repetto D, Camera P, Melani R, Morello N, Russo I, Calcagno E*, et al*. p140Cap regulates memory and synaptic plasticity through Src-mediated and citron-N-mediated actin reorganization. *J Neurosci* 2014; **34**(4)**:** 1542-1553.

30. Sachs NA, Sawa A, Holmes SE, Ross CA, DeLisi LE, Margolis RL. A frameshift mutation in Disrupted in Schizophrenia 1 in an American family with schizophrenia and schizoaffective disorder. *Mol Psychiatry* 2005; **10**(8)**:** 758-764.

31. Carlisle HJ, Luong TN, Medina-Marino A, Schenker L, Khorosheva E, Indersmitten T*, et al*. Deletion of densin-180 results in abnormal behaviors associated with mental illness and reduces mGluR5 and DISC1 in the postsynaptic density fraction. *J Neurosci* 2011; **31**(45)**:** 16194-16207.

32. Cheah PS, Ramshaw HS, Thomas PQ, Toyo-Oka K, Xu X, Martin S*, et al*. Neurodevelopmental and neuropsychiatric behaviour defects arise from 14-3-3zeta deficiency. *Mol Psychiatry* 2012; **17**(4)**:** 451-466.

33. Levchenko A, Davtian S, Freylichman O, Zagrivnaya M, Kostareva A, Malashichev Y. Beta-catenin in schizophrenia: Possibly deleterious novel mutation. *Psychiatry Res* 2015; **228**(3)**:** 843-848.

34. Turner TN, Sharma K, Oh EC, Liu YP, Collins RL, Sosa MX*, et al*. Loss of delta-catenin function in severe autism. *Nature* 2015; **520**(7545)**:** 51-56.

35. Jamain S, Quach H, Betancur C, Rastam M, Colineaux C, Gillberg IC*, et al*. Mutations of the X-linked genes encoding neuroligins NLGN3 and NLGN4 are associated with autism. *Nat Genet* 2003; **34**(1)**:** 27-29.

36. Hamdan FF, Gauthier J, Araki Y, Lin DT, Yoshizawa Y, Higashi K*, et al*. Excess of de novo deleterious mutations in genes associated with glutamatergic systems in nonsyndromic intellectual disability. *Am J Hum Genet* 2011; **88**(3)**:** 306-316.

37. Ikeda Y, Dick KA, Weatherspoon MR, Gincel D, Armbrust KR, Dalton JC*, et al*. Spectrin mutations cause spinocerebellar ataxia type 5. *Nat Genet* 2006; **38**(2)**:** 184-190.

38. Riviere JB, van Bon BW, Hoischen A, Kholmanskikh SS, O'Roak BJ, Gilissen C*, et al*. De novo mutations in the actin genes ACTB and ACTG1 cause Baraitser-Winter syndrome. *Nat Genet* 2012; **44**(4)**:** 440-444, S441-442.

39. Isrie M, Breuss M, Tian G, Hansen AH, Cristofoli F, Morandell J*, et al*. Mutations in Either TUBB or MAPRE2 Cause Circumferential Skin Creases Kunze Type. *Am J Hum Genet* 2015; **97**(6)**:** 790-800.

40. Hutton M, Lendon CL, Rizzu P, Baker M, Froelich S, Houlden H*, et al*. Association of missense and 5'-splice-site mutations in tau with the inherited dementia FTDP-17. *Nature* 1998; **393**(6686)**:** 702-705.

| **Yuan et al. Supplemental Table S2: Neurofilament Light Subunit Protein Expression in Schizophrenia** | | | | |
| --- | --- | --- | --- | --- |
| **Degree of Reduction** | **Statistically Significance** | **Brain Regions** | **Methods** | **References** |
| 50% | Significant | Dorsolateral prefrontal cortex | Western Blot | 1 |
| 40% | Not Significant | Anterior cingulate cortex | Western Blot | 1 |
| 1.7-fold | Significant | Anterior cingulate cortex (white matter) | Proteomics | 2 |
| 2.7-fold | Significant | Corpus callosum | Proteomics | 3 |
| 1.5-fold | Significant | Dorsolateral prefrontal cortex (gray matter) | Proteomics | 4 |
| 1.3-fold | Significant | Dorsolateral prefrontal cortex (white matter) | Proteomics | 5 |
| 2.1-fold | Significant | Dorsolateral prefrontal cortex (gray matter) | Proteomics | 6 |
| 3.1-fold | Significant | Anterior temporal lobe | Proteomics | 7 |
| Significant reduction | Significant | Hippocampus | Proteomics | 8 |
| Significant reduction | Significant | Anterior prefrontal cortex | Proteomics | 9 |
| 3.4-fold | Significant | Corpus callosum | Proteomics | 10 |

**References for Supplemental Table S2**

1. Kristiansen LV, Beneyto M, Haroutunian V, Meador-Woodruff JH. Changes in NMDA receptor subunits and interacting PSD proteins in dorsolateral prefrontal and anterior cingulate cortex indicate abnormal regional expression in schizophrenia. *Molecular psychiatry* 2006; **11**(8)**:** 737-747, 705.

2. Clark D, Dedova I, Cordwell S, Matsumoto I. Altered proteins of the anterior cingulate cortex white matter proteome in schizophrenia. *Proteomics Clin Appl* 2007; **1**(2)**:** 157-166.

3. Sivagnanasundaram S, Crossett B, Dedova I, Cordwell S, Matsumoto I. Abnormal pathways in the genu of the corpus callosum in schizophrenia pathogenesis: a proteome study. *Proteomics Clin Appl* 2007; **1**(10)**:** 1291-1305.

4. Pennington K, Beasley CL, Dicker P, Fagan A, English J, Pariante CM*, et al*. Prominent synaptic and metabolic abnormalities revealed by proteomic analysis of the dorsolateral prefrontal cortex in schizophrenia and bipolar disorder. *Mol Psychiatry* 2008; **13**(12)**:** 1102-1117.

5. English JA, Dicker P, Focking M, Dunn MJ, Cotter DR. 2-D DIGE analysis implicates cytoskeletal abnormalities in psychiatric disease. *Proteomics* 2009; **9**(12)**:** 3368-3382.

6. Martins-de-Souza D, Gattaz WF, Schmitt A, Maccarrone G, Hunyadi-Gulyas E, Eberlin MN*, et al*. Proteomic analysis of dorsolateral prefrontal cortex indicates the involvement of cytoskeleton, oligodendrocyte, energy metabolism and new potential markers in schizophrenia. *Journal of psychiatric research* 2009; **43**(11)**:** 978-986.

7. Martins-de-Souza D, Gattaz WF, Schmitt A, Rewerts C, Marangoni S, Novello JC*, et al*. Alterations in oligodendrocyte proteins, calcium homeostasis and new potential markers in schizophrenia anterior temporal lobe are revealed by shotgun proteome analysis. *Journal of neural transmission* 2009; **116**(3)**:** 275-289.

8. Focking M, Dicker P, English JA, Schubert KO, Dunn MJ, Cotter DR. Common proteomic changes in the hippocampus in schizophrenia and bipolar disorder and particular evidence for involvement of cornu ammonis regions 2 and 3. *Archives of general psychiatry* 2011; **68**(5)**:** 477-488.

9. Wesseling H, Gottschalk MG, Bahn S. Targeted multiplexed selected reaction monitoring analysis evaluates protein expression changes of molecular risk factors for major psychiatric disorders. *The international journal of neuropsychopharmacology* 2014; **18**(1).

10. Saia-Cereda VM, Cassoli JS, Schmitt A, Falkai P, Nascimento JM, Martins-de-Souza D. Proteomics of the corpus callosum unravel pivotal players in the dysfunction of cell signaling, structure, and myelination in schizophrenia brains. *European archives of psychiatry and clinical neuroscience* 2015; **265**(7)**:** 601-612.
