# Supplementary figures and images for: Neurofilament light interaction with GluN1 modulates neurotransmission and schizophrenia-associated behaviors
Source: Transl Psychiatry. 2018 Aug 24;8:167. doi: 10.1038/s41398-018-0194-7 (PMC6109052; doi:10.1038/s41398-018-0194-7)

Yuan et al. Supplemental Figure S1

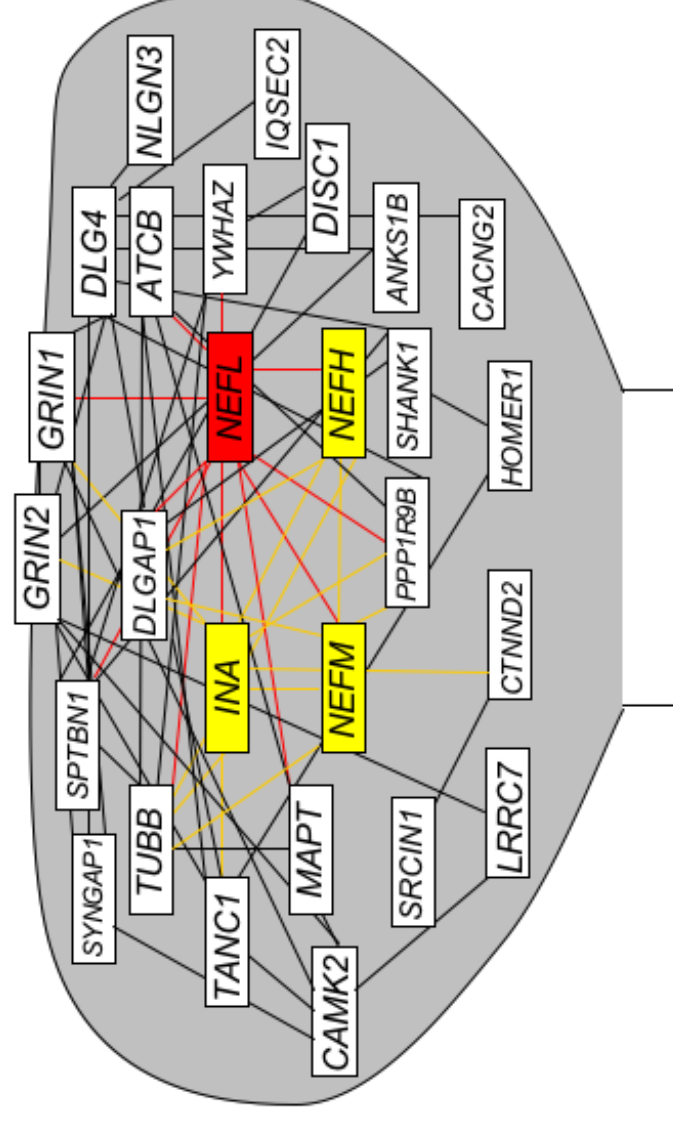

Supplement: Supplementary file 2 — Supplemental Figure S1 [file 41398_2018_194_MOESM2_ESM.pdf]

## Yuan et al. Supplemental Figure S2

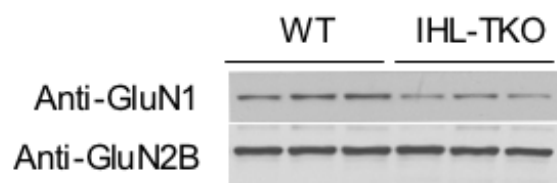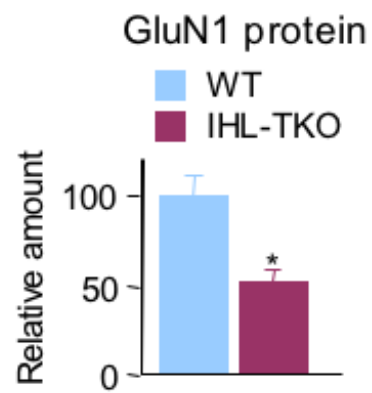

Supplement: Supplementary file 3 — Supplemental Figure S2 [file 41398_2018_194_MOESM3_ESM.pdf]

Yuan et al. Supplemental Figure S3

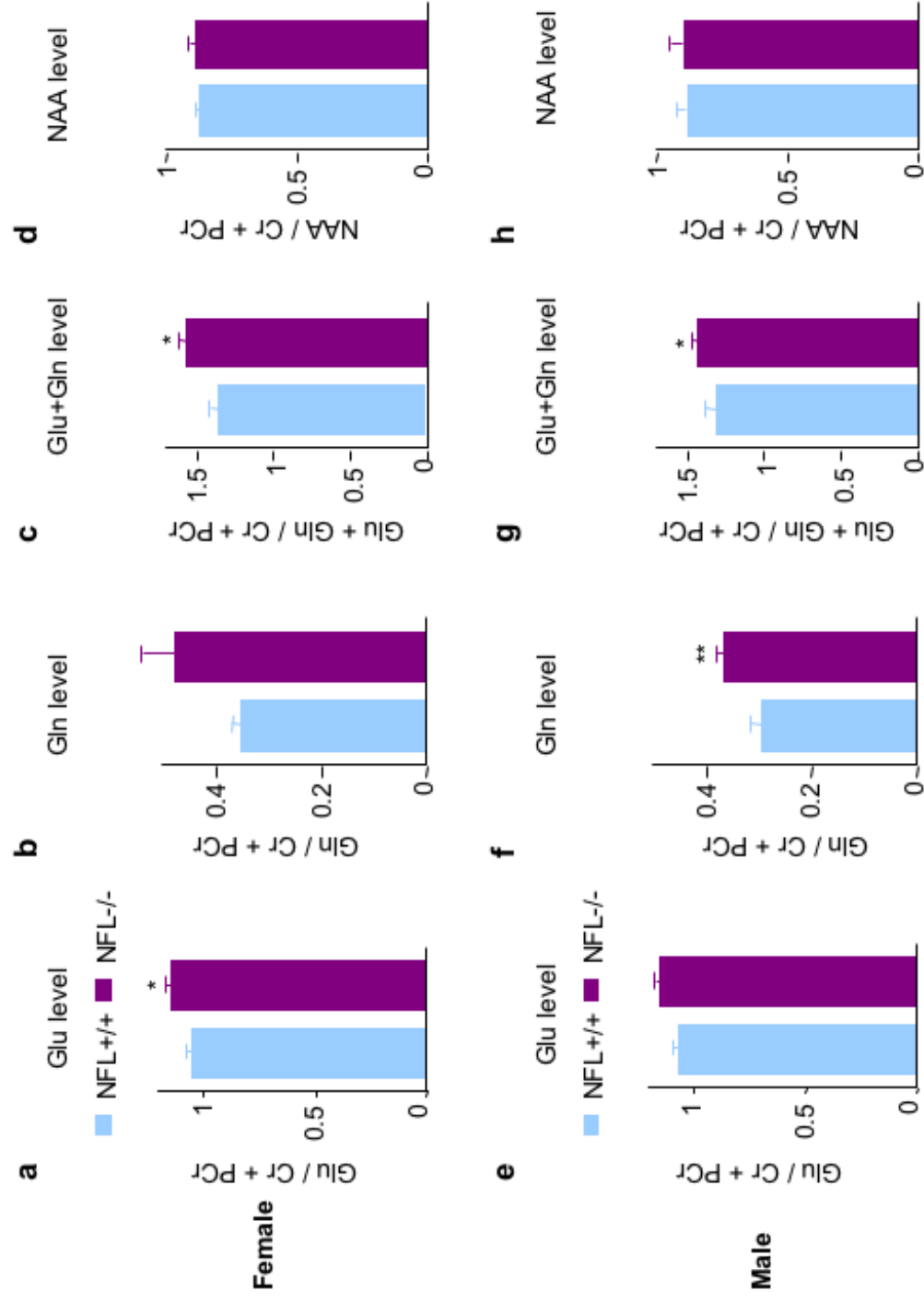

Supplement: Supplementary file 4 — Supplemental Figure S3 [file 41398_2018_194_MOESM4_ESM.pdf]

Yuan et al. Supplemental Figure S4

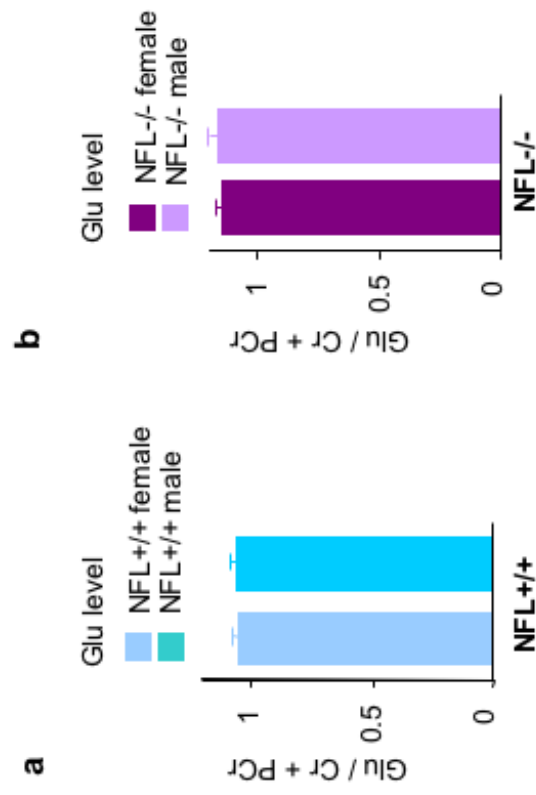

Supplement: Supplementary file 5 — Supplemental Figure S4 [file 41398_2018_194_MOESM5_ESM.pdf]

**Yuan et al. Supplemental Figure S5**

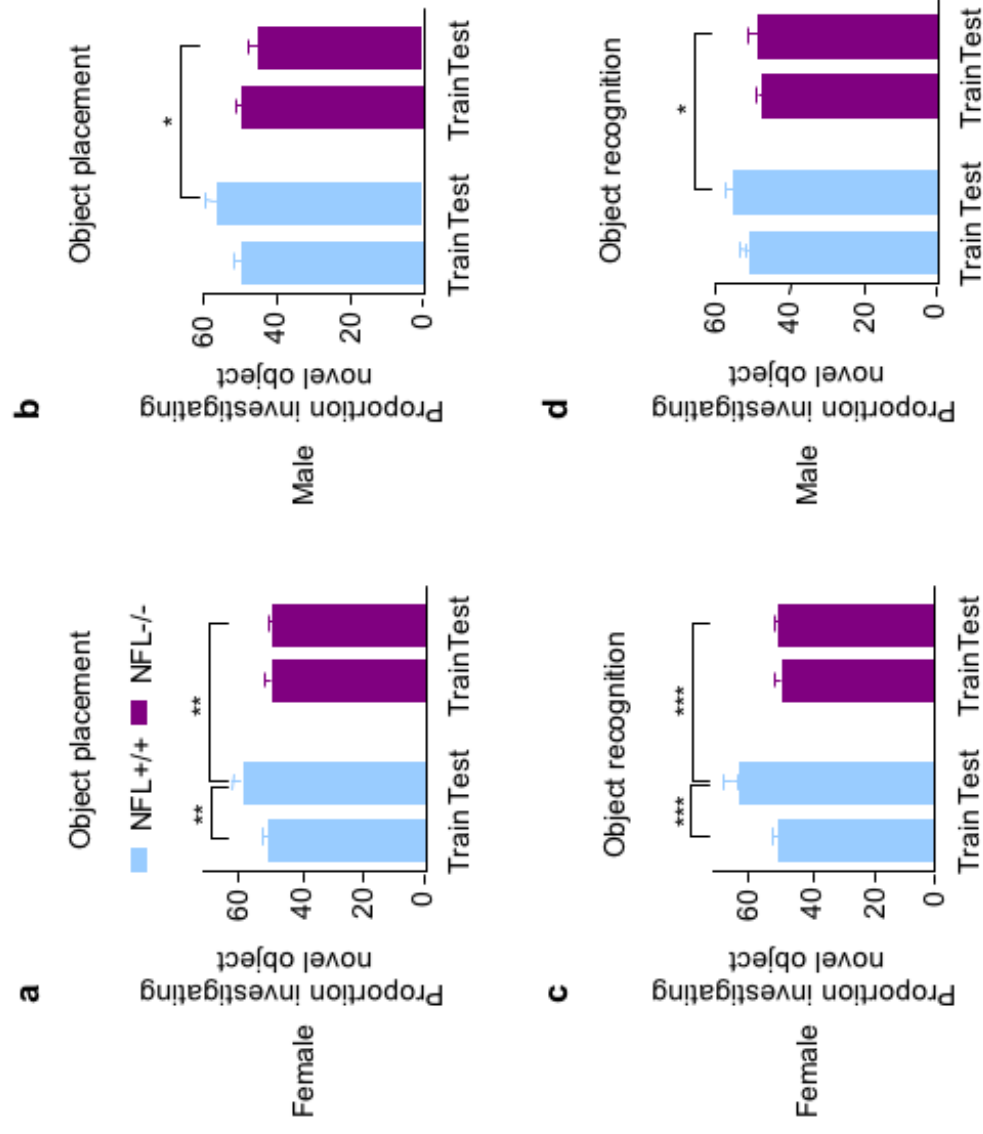

Supplement: Supplementary file 6 — Supplemental Figure S5 [file 41398_2018_194_MOESM6_ESM.pdf]

## Yuan et al. Supplemental Figure S6

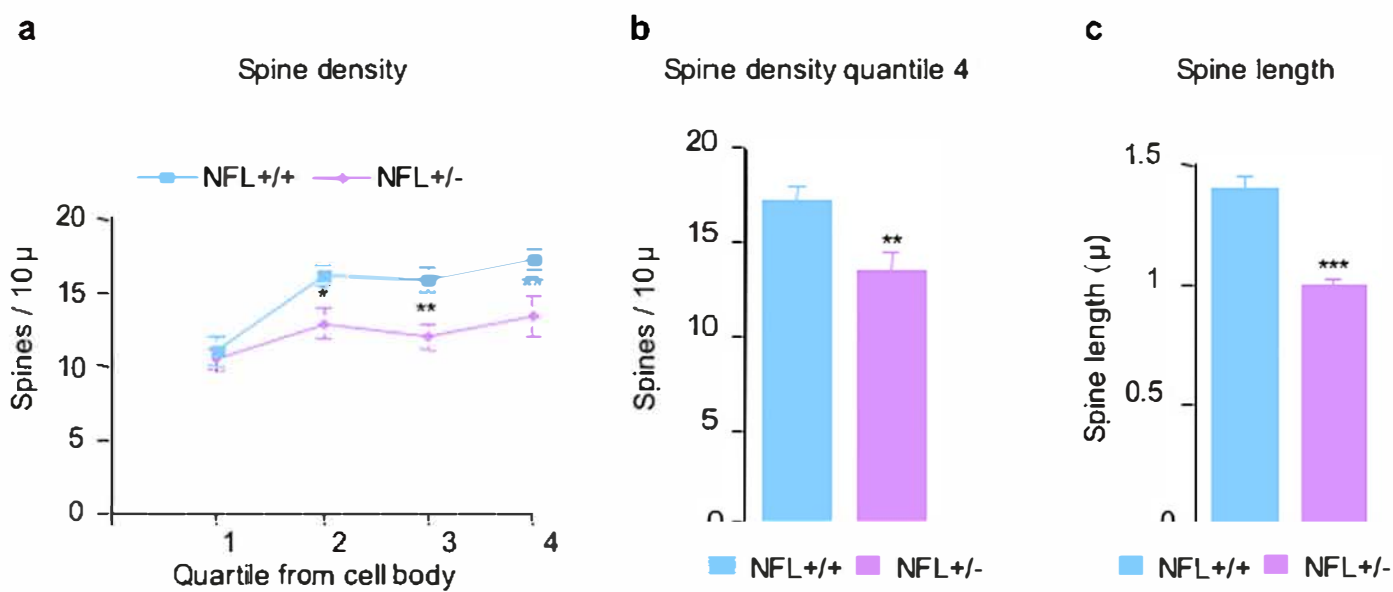

Supplement: Supplementary file 7 — Supplemental Figure S6 [file 41398_2018_194_MOESM7_ESM.pdf]
