# Supplementary material for: Neurofilament light interaction with GluN1 modulates neurotransmission and schizophrenia-associated behaviors
Source: Transl Psychiatry. 2018 Aug 24;8:167. doi: 10.1038/s41398-018-0194-7 (PMC6109052; doi:10.1038/s41398-018-0194-7)
Supplement: Supplementary file 8 — Supplemental Table S3 [file 41398_2018_194_MOESM8_ESM.pdf]

# Yuan et al. Supplemental Table S3

**Change of dendrite length in dentate gyrus granule cells of NFL -/- mice**

| | NFL+/+ | NFL+/+ SE | NFL-/- | NFL -/- SE | p-value (T-test) |
|---|---|---|---|---|---|
| Total dendrite length mesured | 983 | 58 | 827 | 81 | 0.141 |
| | | | | | |
| Number of branch points | 5.3 | 0.3 | 5.9 | 0.67 | 0.402 |
| Total length of non-terminal segments | 150 | 19 | 148 | 18.8 | 0.951 |
| Average length of non-terminal segments | 27 | 2.56 | 25 | 2.05 | 0.417 |
| | | | | | |
| Number of natural endings | 4.1 | 0.29 | 3.4 | 0.26 | 0.219 |
| Total length of natural end terminal segments | 622 | 49 | 438 | 39.46 | 0.021 |
| Average length of natural end terminal segments | 155 | 5.94 | 129 | 6.25 | 0.013 |
| | | | | | |
| Number of truncated ending | 2.8 | 0.29 | 3.8 | 0.64 | 0.154 |
| Total length of truncated end terminal segments | 219 | 33 | 216 | 41.42 | 0.955 |
| Average number of truncated end segments | 76 | 6.05 | 59 | 8.06 | 0.15 |
| | | | | | |
| Average path length | 207 | 4.65 | 181 | 5.44 | 0.004 |
| Maximum path length | 227 | 7.09 | 195 | 7.94 | 0.023 |
